# Decoding Cellular Dynamics in Epidermal Growth Factor Signaling Using a New Pathway-Based Integration Approach for Proteomics and Transcriptomics Data

**DOI:** 10.3389/fgene.2015.00351

**Published:** 2016-01-07

**Authors:** Astrid Wachter, Tim Beißbarth

**Affiliations:** Department of Medical Statistics, University Medical CenterGöttingen, Germany

**Keywords:** omics, data integration, high-throughput, time-series, EGF signaling

## Abstract

Identification of dynamic signaling mechanisms on different cellular layers is now facilitated as the increased usage of various high-throughput techniques goes along with decreasing costs for individual experiments. A lot of these signaling mechanisms are known to be coordinated by their dynamics, turning time-course data sets into valuable information sources for inference of regulatory mechanisms. However, the combined analysis of parallel time-course measurements from different high-throughput platforms still constitutes a major challenge requiring sophisticated bioinformatic tools in order to ease biological interpretation. We developed a new pathway-based integration approach for the analysis of coupled omics time-series data, which we implemented in the R package *pwOmics*. Unlike many other approaches, our approach acknowledges the role of the different cellular layers of measurement and infers consensus profiles and time profile clusters for further biological interpretation. We investigated a time-course data set on epidermal growth factor stimulation of human mammary epithelial cells generated on the two layers of RNA and proteins. The data was analyzed using our new approach with a focus on feedback signaling and pathway crosstalk. We could confirm known regulatory patterns relevant in the physiological cellular response to epidermal growth factor stimulation as well as identify interesting new interactions in this signaling context, such as the regulatory influence of the connective tissue growth factor on transferrin receptor or the influence of growth arrest and DNA-damage-inducible alpha on the connective tissue growth factor. Thus, we show that integrated cross-platform analysis provides a deeper understanding of regulatory signaling mechanisms. Combined with time-course information it enables the characterization of dynamic signaling processes and leads to the identification of important regulatory interactions which might be dysregulated in disease with adverse effects.

## Introduction

Omics data integration is a conclusive concept for a systemic understanding of biological signaling mechanisms, both in healthy conditions and disease (Kristensen et al., 2014; Ritchie et al., 2015). The combination of different types of omics data can provide a more comprehensive and complete picture of individual cellular mechanisms. Furthermore, a cross-platform analysis represents a measure to overcome individual platform biases and technical limitations (Yeger-Lotem et al., 2009).

**Figure 1:**
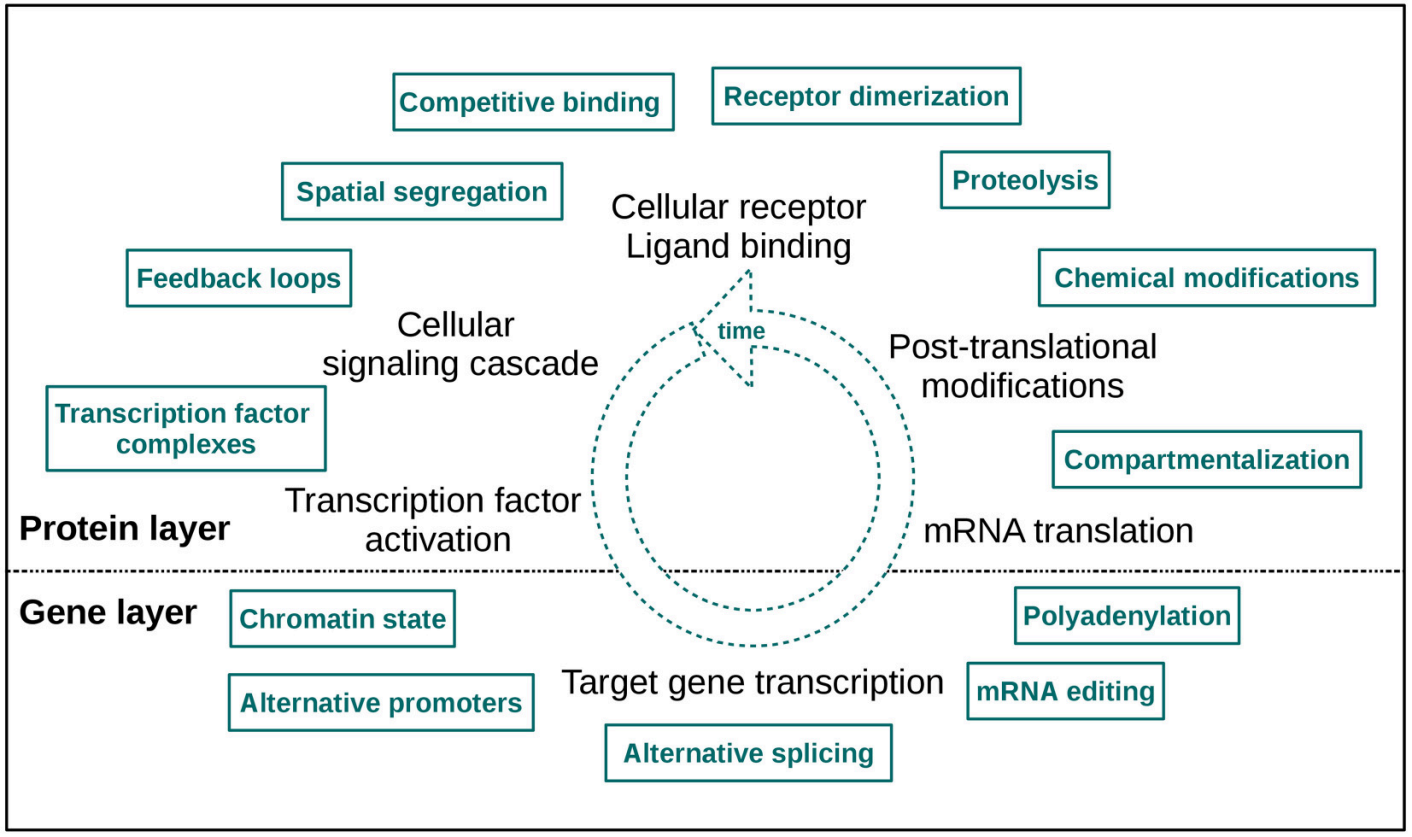
**A selection of cellular layer specific regulatory signaling mechanisms**. The two layers of measurement are indicated as “protein” and “gene layer.” The high number of effectors illustrates the mechanistic fine-tuning of signaling. Note that this fine-tuning also takes place in the dimension of time.

An even more informative approach is to analyze time-course data sets from different omics levels, as a lot of cellular signaling information is encoded in signaling dynamics (Purvis and Lahav, 2013). This type of data provides more than only a single “snapshot” of the underlying biological processes, thus it can augment the knowledge we have about cellular signaling events considerably. With these data feedback signaling loops, molecular interactions and pathway crosstalk can be tracked over time. Thus, combining different types of omics data with time course information enables a comprehensive characterization of cellular responses upon stimulation and also a detection of regulatory mechanisms initiated by specific perturbations. In Figure 1 a selection of dynamic regulatory signaling mechanisms on protein and gene layer is depicted. These effects become directly apparent in such omics data sets, so the “dynamic knowledge” we can collect may also provide us with an idea of modifications responsible for pathologic signaling and signaling dynamics, thus forming a basis for an improvement of treatment strategies.

Of course, such parallel time-course data sets are even more challenging to analyze and interpret as they include an additional dimension and require a meaningful cross-platform integration method. Hence, there is a demand for bioinformatic tools that can deal with the diverse data types and combine them in such a way that their output enables a straightforward biological interpretation of the data. Although a lot of individual data integration methods have been developed so far, they mostly address very specific integration questions (Balbin et al., 2013; Hamon et al., 2014), are not implemented as tools which can be freely used by other biologists and bioinformaticians [e.g., QIAGEN's Ingenuity® Pathway Analysis (IPA®, QIAGEN Redwood City^1^)] or do not acknowledge the different nature of different omics data types (Ding et al., 2012; Sun et al., 2014). Very few tools also include the biologically very interesting aspect of time-course data analysis (Rogers et al., 2008), although these types of data sets are expected to be generated more often in the near future (Bar-Joseph et al., 2012) in order to address systems biology questions.

We developed a pathway-based data integration approach for the analysis of coupled high-throughput time-course measurements on the cellular layers of proteins, transcripts and genes. We implemented this approach as R package *pwOmics*, that we presented earlier (Wachter and Beissbarth, 2015). In brief, *pwOmics* joins the tools of network analysis: It uses public signaling pathway knowledge to map molecular network interactions, thereby identifying activated and inactivated genes and proteins in cellular signaling upon perturbation. Thus, the cellular layers on which the data is collected are acknowledged during data analysis while simultaneously considering the dynamics. Here we describe and test the utility of our method in more detail.

**Figure 2:**
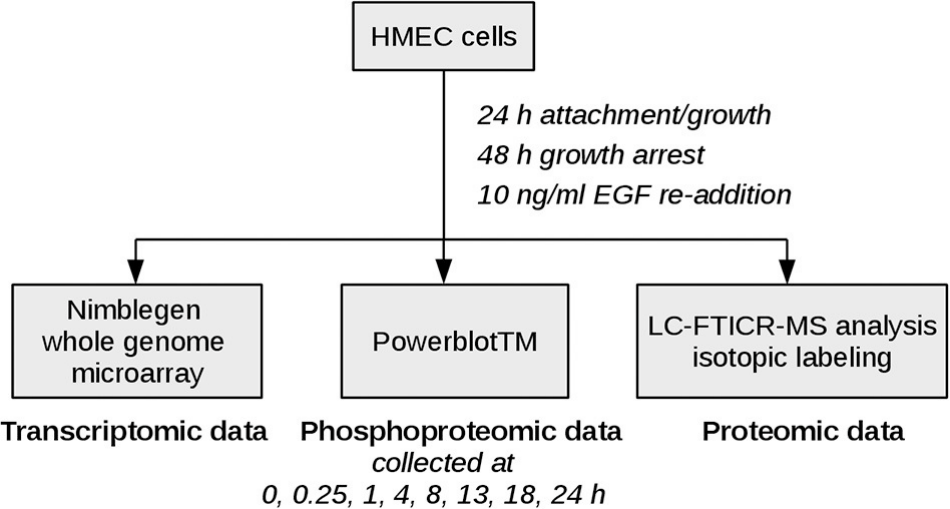
**Experimental design**. HMEC cells were seeded and allowed to attach and grow for 24 h. After 48 h of growth arrest with medium lacking serum, EGF and other growth factors, EGF was added again to monitor the mitogenic response of the cells. Samples for high-throughput genomic and proteomic measurements were taken at time points 0, 0.25, 1, 4, 8, 13, 18, 24 h after EGF stimulation. The 0.25 h time point was excluded from the microarray data set due to quality issues, therefore the coupled data set on which our analysis is based includes time points 0, 1, 4, 8, 13, 18, and 24 h after EGF stimulation.

Epidermal growth factor (EGF) signaling has already been studied comprehensively in comparison to other signaling pathways as dysregulation is associated with poor prognosis in many human malignancies (Lurje and Lenz, 2009). As various high-throughput and low-throughput omics data sets are available and a lot of knowledge is already acquired on the basis of which methodical evaluation can be performed, it constitutes an adequate example for investigation of new approaches. The data set analyzed here measures the mitogenic response of human mammary epithelial cells (HMEC) to EGF on the proteomic and the transcriptomic layer over time (Waters et al., 2012), thereby representing physiological signaling conditions. Figure 2 depicts the experimental design used in the study. EGF stimulation is associated with cellular proliferation, differentiation and survival (Herbst, 2004) and directly affects signaling pathways such as the MAPK signaling pathway, the ERBB signaling pathway and the RAS signaling pathway.

We chose the comparably well characterized example of EGF signaling in order to map the results of our new pathway-based integration approach to known experimental results for methodical evaluation and to reveal new dynamically relevant mechanisms in EGF signaling on the different functional layers. We focus on feedback signaling and pathway crosstalk, both complex regulatory mechanisms that have been under intensive biological investigation in individual experiments in physiological and pathological conditions (Avraham and Yarden, 2011; Wang et al., 2011).

## Methods

### Data set

The data set investigated with the new pathway-based integration approach was generated in a study on network analysis of EGF signaling. The experimental design used is illustrated in Figure 2, the measurements included transcriptomic, proteomic and phosphoproteomic data generation. Further details as well as the preprocessing steps performed on both microarray raw data and proteomic raw data are described in Waters et al. (2012). The raw microarray data files are available via the Gene Expression Omnibus database, GSE15668 (Waters et al., 2012). The corresponding proteomic data is also publicly available^2^.

Shortly, biological samples were hybridized against NimbleGen microarrays. A quality check revealed that time point 0.25 h failed to hybridize, therefore the coupled data set analyzed here includes only time points 0, 1, 4, 8, 13, 18, and 24 h after EGF stimulation. Proteome analysis was performed MS-based, while phosphoproteome data were collected as part of a parallel western blot analysis. For each time point differentially expressed transcripts or differentially abundant phosphoproteins/proteins compared to time point 0 h were determined. Raw microarray data was quantile normalized before performing a pairwise analysis of variance with a 5% false discovery rate to determine differentially expressed transcripts. Proteome and phosphoproteome levels were considered significant when passing specific quality checks and showing a fold change ≥1.5.

### Databases

Pathway information used for the pathway-based integration approach were taken from KEGG (Kanehisa and Goto, 2000; Kanehisa et al., 2014), Reactome (Croft et al., 2014), Pathway Interaction Database (Schaefer et al., 2009), and Biocarta (Nishimura, 2001). This information was used as gene sets in the analysis of the phosphoproteome data and combined with its topological information in the transcriptome data analysis. It was downloaded via the AnnotationHub R package^3^ from Bioconductor (Huber et al., 2015) as BioPAX level 2 files and then processed further with the rBiopaxParser R package (Kramer et al., 2013). The transcription factor (TF)—target gene interaction information from the TRANSFAC® database (Biobase version 2014.4; Matys et al., 2006) was used. Network reconstruction was based on the connected protein-protein interaction (PPI) network of the STRING database (Franceschini et al., 2013).

### Analyses

**Figure 3:**
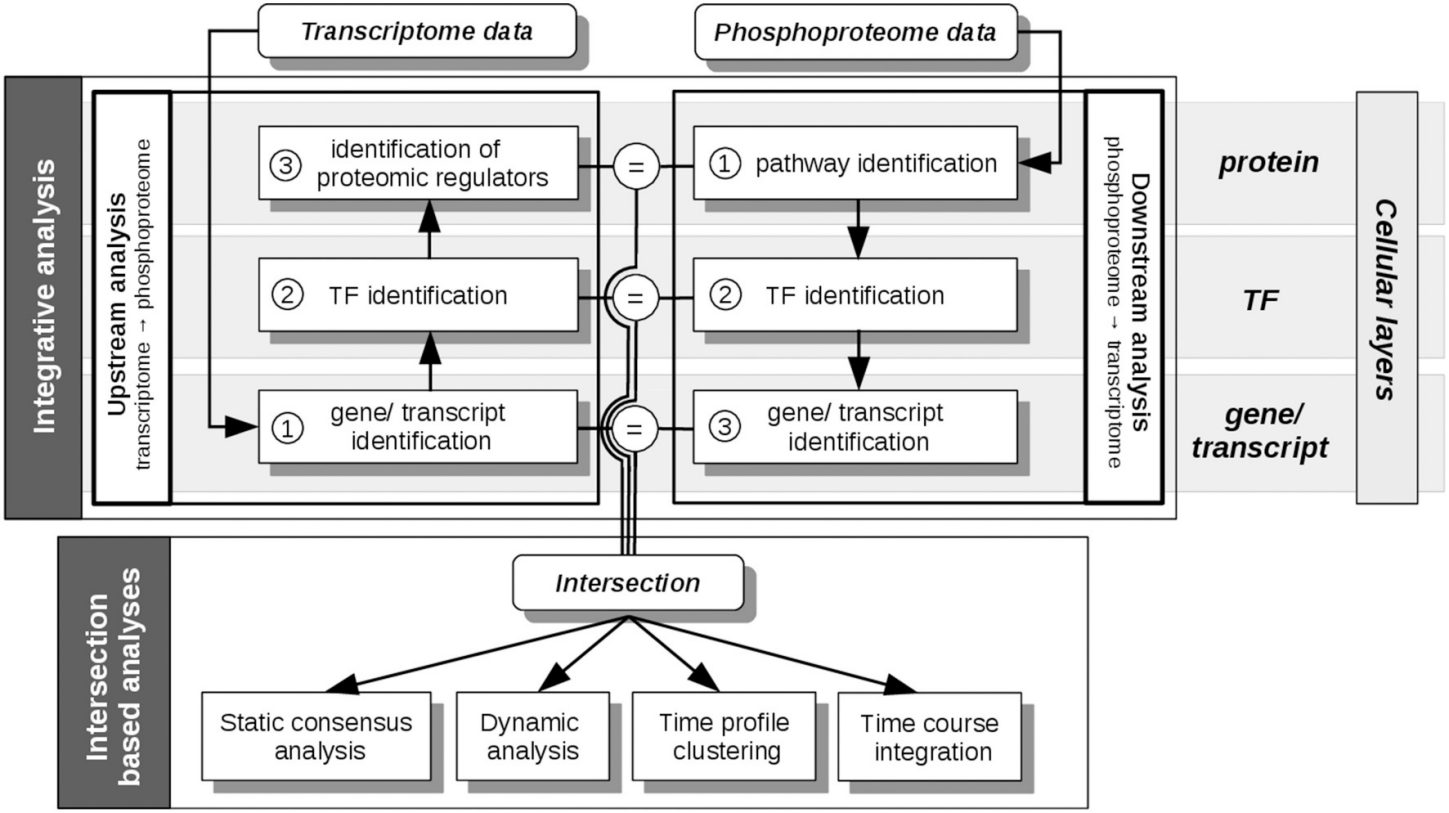
**pwOmics analyses steps**. In the initial integrative analysis a downstream analysis of the phosphoproteome data and an upstream analysis of the transcriptome data is performed. The former includes the identification of pathways that include differentially abundant phosphoproteins, the identification of the TFs in these pathways and the determination of downstream target genes. In the upstream analysis the differentially expressed transcripts are identified, as well as their upstream TFs. By determining the pathways of these TFs also potential proteomic regulators can be identified. The intersection of the molecules on each cellular layer (protein, TF and gene/transcript) is determined before the intersection based analyses are performed. These include a static consensus analysis that can be performed for each measured time point, the consensus-based dynamic analysis that enables the generation of a probabilistic network exploiting the time-course information of those molecules that are part of the consensus analysis result. Furthermore, in a time profile clustering co-regulation patterns can be identified. Eventually, the time course integration allows to map downstream consensus transcripts with differentially abundant proteins. The “=” sign depicts the molecular overlap on each cellular layer, corresponding to the layer-specific consensus molecules.

**Figure 4:**
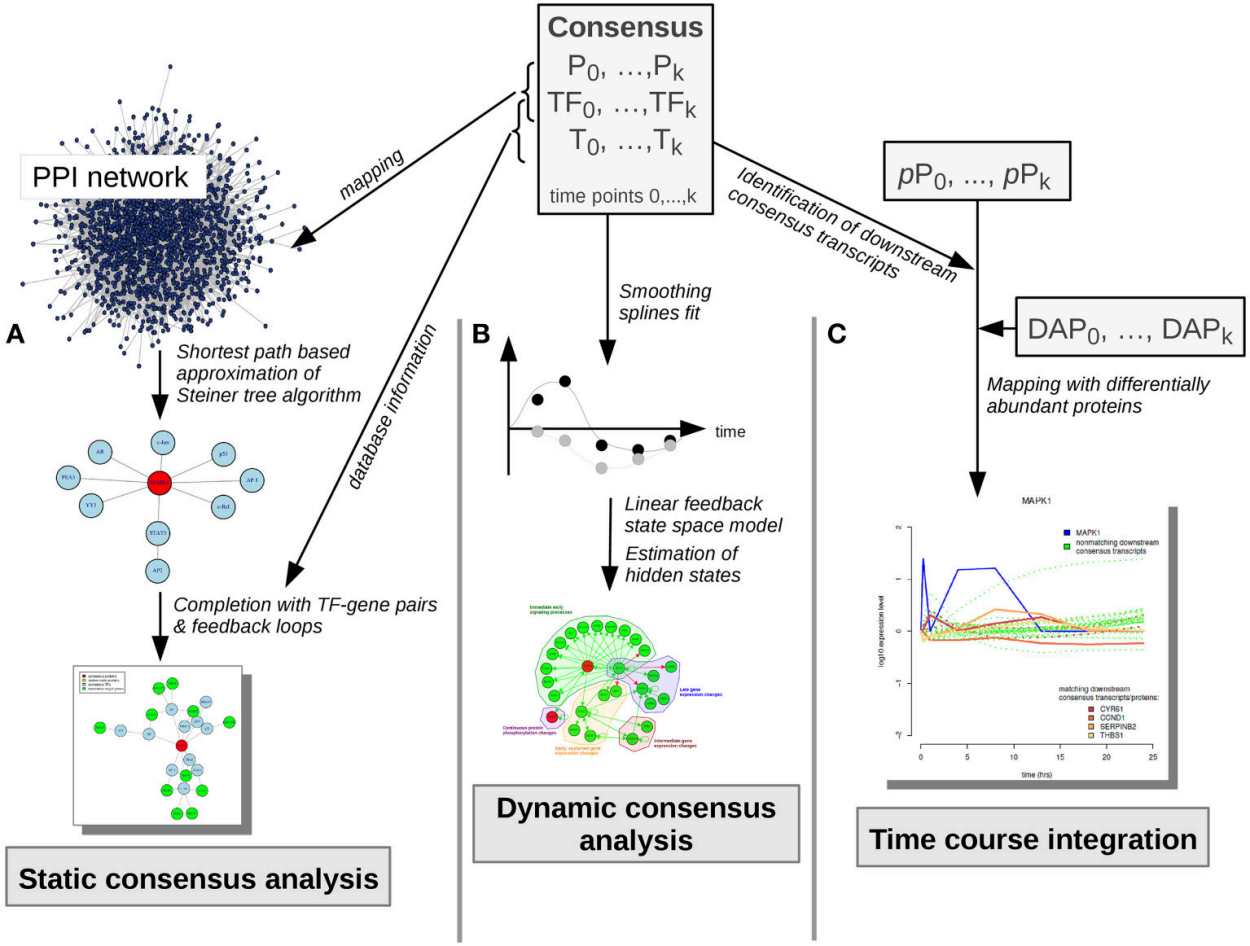
**Consensus molecule analyses**. Consensus molecules on each cellular layer are used for the static consensus analysis, the dynamic consensus analysis and the time-course integration. **(A)** In the static consensus analysis static graphs are generated based on the PPI-mapped consensus proteins and transcription factors, an approximation of the Steiner tree algorithm is applied and the connected networks are complemented with TF-target interactions from TRANSFAC database. In case both consensus gene and corresponding consensus protein are part of the network, feedback loops are added. **(B)** In the dynamic consensus analysis smoothing splines are fitted to the time courses for all consensus molecules. Based on the higher density data set a linear feedback state space model is generated, hidden states are estimated and a probabilistic network is generated with dynamic Bayesian network inference (ebdbNet R package). **(C)** In the time course integration downstream consensus transcripts of the differentially abundant phosphoproteins are identified. These are mapped to the differentially abundant proteins. Time-courses of the downstream signaling players are visualized, subsequently. P, consensus proteins; TF, consensus transcription factors; T, consensus transcripts; pP, phosphoproteins; DAP, differentially abundant proteins.

All analysis steps described here are based on pre-processed transcriptome, proteome and phosphoproteome data, as described in Waters et al. (2012). Main analyses steps were performed with the R package *pwOmics* (Wachter and Beissbarth, 2015). Our methodical framework is depicted in Figures 3, 4.

#### Data processing

First, individual analyses of the omics data sets were performed during phosphoprotein data based downstream and transcript based upstream analysis (Figure 3). For the downstream analysis an identification of the pathways, which include differentially abundant phosphoproteins, was performed. The transcription factors of these pathways were then found by matching the gene sets of the pathways against the transcription factors listed in the transcription factor—target gene database. Downstream target genes were identified, equivalently. The downstream analysis is based in general on the assumption of downstream regulation upon protein phosphorylation. Upstream analysis identified the upstream TFs of significantly differentially regulated transcripts. Subsequently, pathways including these TFs were identified in order to find possible upstream proteomic regulators of differentially expressed transcripts. The parameters chosen here corresponded to at least one TF per pathway for pathway identification and 10 orders of neighbors identified upstream of the TF for potential proteomic regulators. The results of each functional layer of signaling (pathway layer, TF layer, and gene/transcript layer) of downstream and upstream analysis were compared. These analyses steps were performed for each time point. Gene and protein ID matching was done by conversion of all IDs to HUGO gene symbols.

#### Static consensus analysis

In the static consensus analysis integrated signaling networks were constructed based on intersecting proteins, TFs, genes and transcripts on each functional layer (Figure 4A). The consensus proteins and TFs were mapped to the PPI STRING database and Steiner trees were generated via a shortest paths based approximation algorithm (Sadeghi and Fröhlich, 2013). The graphs were then completed by adding the corresponding TF—target interactions using TRANSFAC information. In case both consensus gene and consensus protein were part of the static consensus graph feedback loops were added.

#### Dynamic consensus analysis

In order to leverage the complete dynamic information from the data sets dynamic analysis was performed on basis of all consensus molecules (Figure 4B). The data associated with these nodes was used to fit cubic smoothing splines in order to generate a sufficiently dense data set for network inference via empirical Bayes estimation of a dynamic bayesian network with the R package ebdbNet (Rau et al., 2010). The generation of data points was based on the simplifying assumption of a gradual change of signaling over time. For further parameters default values were chosen. For visualization of the dynamic bayesian network a probability threshold was chosen which reflects a moderate number of regulatory interactions with a high probability in the network. The resulting threshold for plotting of the edges corresponded to a probability of an edge to be present by chance of 0.15.

#### Time profile clustering

Additionally, time profile clustering was performed in order to identify co-regulation patterns: Combining the described integration approach with a soft clustering implemented as fuzzy c-means algorithm (Kumar and Futschik, 2007) yielded an integrated time profile clustering based on the log-fold changes of consensus proteins and transcripts.

#### Time course integration

For further time course based integration with the proteome data set downstream consensus transcripts of the measured phosphoproteins were determined (Figure 4C). In a next step these were mapped to proteins, that were significantly differentially abundant at any time point (Figure 2, proteomic data).

## Results

### Individual downstream and upstream analyses

We performed individual downstream and upstream analyses of the phosphoproteome and microarray data sets taking into account the different functional layers of the cell the data originates from. The used pathway information exploits the signaling knowledge stored in public databases. Figure 3 illustrates the steps of the individual analyses and further analysis steps explained in the next sections. Table 1 shows the corresponding numbers of identified molecules and pathways on the different functional cellular layers in downstream and upstream analysis.

**Table 1:** **Individual analysis**.

**Time after EGF stimulation [h]**	**0.25**	**1**	**4**	**8**	**13**	**18**	**24**
**DOWNSTREAM ANALYSIS**							
No. of differentially abundant phosphoproteins	5	3	3	2	3	2	2
No. of pathways	121	68	98	90	81	79	79
No. of TFs	64	61	62	62	62	62	62
No. of potential target genes	1296	1293	1294	1294	1295	1295	1295
**UPSTREAM ANALYSIS**							
No. of differentially expressed transcripts	−	35	87	66	85	134	1551
No. of TFs	−	140	111	146	199	212	480
No. of pathways	−	163	154	169	200	200	230
No. of potential upstream proteomic regulators	−	871	950	897	920	976	1023

*Downstream and upstream analyses characteristics over time. The expected bottleneck on the transcription factor layer can be observed. In the downstream analysis most pathways are overlapping, so we observe no large difference in the target gene numbers. The pre-processed proteomic data set comprises one time point of measurement more than the transcriptomic data set (0.25 h after EGF stimulation)*.

The data set for the phosphoproteome based downstream analysis is very small with only five phosphoprotein abundances investigated. However, as these were chosen thoroughly in the experiment we observe a considerable number of pathways that are influenced in downstream signaling. Altogether 121 pathways were identified when querying the four pathway databases used for the analysis. However, this set might include partly redundant pathways when originating from different databases, but describing the same signaling pathway. Pathways that are identified in every time point include e.g., the Biocarta “egf signaling” pathway, the NCI “EGF receptor (ErbB1) signaling pathway,” the NCI pathway “EGFR-dependent Endothelin signaling events” or the NCI pathway “ErbB1 downstream signaling.” Furthermore, a number of pathways are identified that are involved in cellular adhesion, STAT3 dependent signaling and PI3K signaling. Differential abundance of phopho-MAPK14 was only identified at time point 0.25 h after EGF stimulation. Corresponding pathways identified for that time point included e.g., the Biocarta “p38 mapk signaling pathway” and the Biocarta “mapkinase signaling pathway.” According to the TF—target gene database the identified TFs activate the expression of a high number of genes as shown in Table 1.

In the transcriptome based upstream analysis an identification of upstream TFs was performed based on the differentially expressed transcripts. Corresponding numbers at each time point after EGF stimulation are displayed in Table 1. Identified upstream pathways included e.g., the “MAPK signaling pathway,” the “EGF receptor (ErbB1) signaling pathway” and the “ErbB1 downstream signaling” pathway. The higher numbers of differentially expressed transcripts resulted likewise in the identification of more pathways. In those pathway sets the topological information enabled the identification of possible upstream proteomic regulators, subsequently.

The pathways identified in the downstream and upstream analyses at each measured time point after EGF stimulation are part of the Supplementary Material (Tables S2, S3).

### Consensus analysis

**Figure 5:**
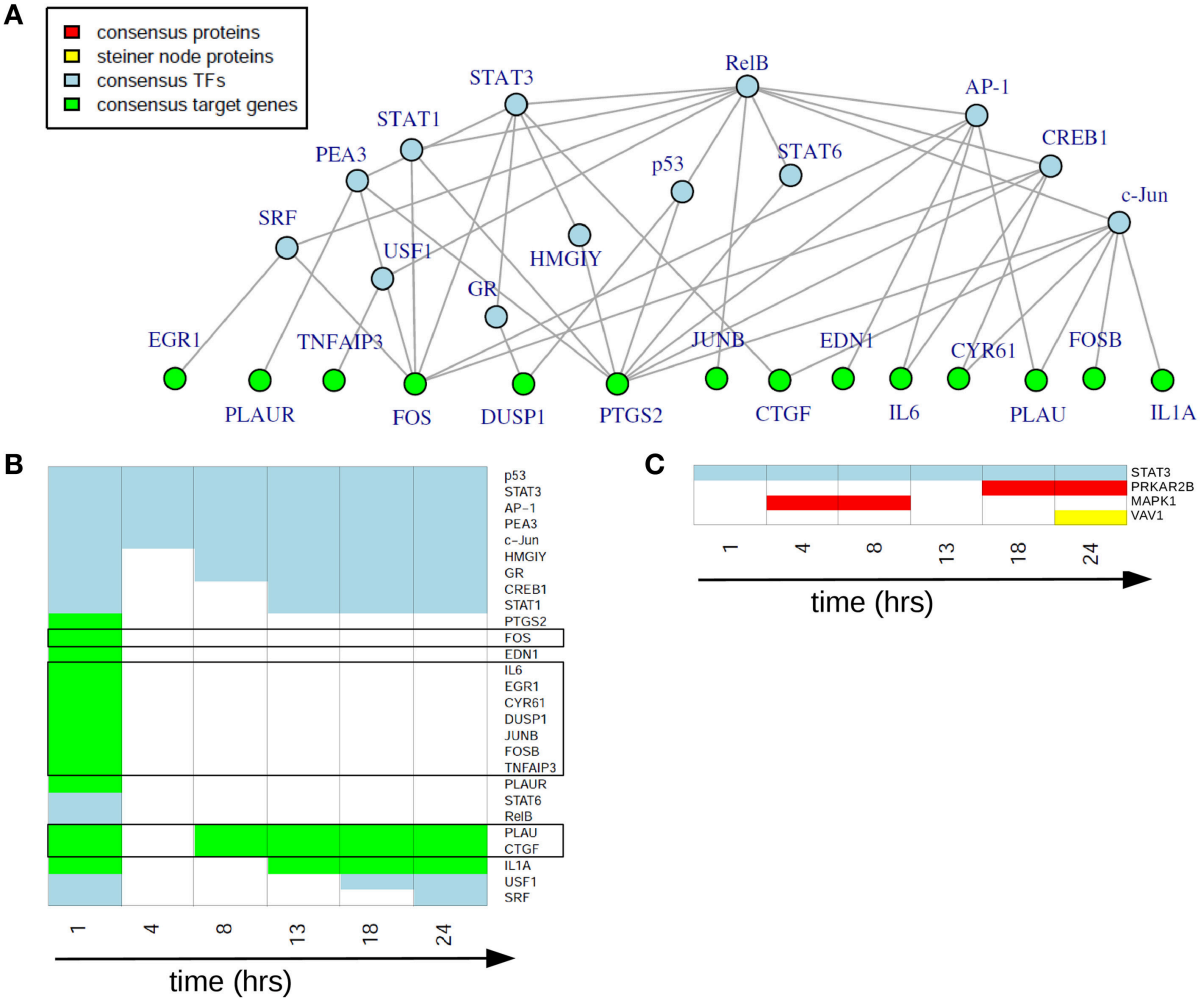
**Static consensus analysis results**. **(A)** Static consensus graph for time point 1 h after EGF stimulation. **(B)** Static consensus profiles for members of the static consensus graph 1 h after EGF stimulation. Colors in the heatmap correspond to colors used in the consensus graphs, “white” boxes represent no membership in the consensus graph at that time point after EGF stimulation. Genes known to be IEGs (according to Tullai et al., 2007) are framed in black. **(C)** Static consensus profiles for selected proteins.

In the static consensus analysis we integrated the results of the different platforms for each time point on each functional layer. The aim was to reduce the individual downstream and upstream analyses results to molecule sets which include those molecules identified from both platforms and to reduce at the same time false positive molecules on the different functional layers. Exemplary, the consensus network of 1 h after EGF stimulation is shown in Figure 5A, later time point static consensus networks are part of the Supplementary Material (Figures S2–S7). These networks provide interaction and regulatory information on the consensus molecules. Yet, in our further analyses we focus on the static consensus profiles reflecting the presence of specific molecules in the consensus networks at each time point, as illustrated in Figure 5B. The static consensus profiles were used to explore the static consensus characteristics of certain molecules in order to evaluate the integration method. As dynamic signaling is especially interesting with regard to feedback signaling mechanisms and pathway crosstalk, we focus on these two signaling patterns in the following. Figure 5B shows the static consensus profiles of the members of the static consensus graph 1 h after EGF stimulation. A considerable number of genes being part of this consensus graph are exclusively found at this early time point. The profiles additionally show that both *PLAU*, the urokinase-type plasminogen activator, and *CTGF*, the connective tissue growth factor, comprise late regulatory changes. A figure with all static consensus profiles is part of the Supplementary Material (Figure S1). In these, 13 of 19 genes that are at least identified at two time points not including the 1 h time point after stimulation show a sustained pattern, indicative of a secondary cellular response. The genes without such a sustained pattern are *PLAU, CTGF* and *IL1A*, being already active 1 h after EGF stimulation or genes showing an intermediate activation.

Next, we investigated the pattern of proteins in the static consensus networks as well as the identified steiner nodes. The first group comprises the intersection of differentially abundant phosphoproteins in the proteomic data set and the potential upstream proteomic regulators of the differentially expressed genes. The second group is derived by generating Steiner trees after mapping the consensus molecules to the PPI network and might be functionally interesting, as its nodes are candidates for the regulation of the unconnected, mapped proteins. The static consensus profiles of the included proteins and the steiner node identified in this analysis are shown in Figure 5C. Transcription factor STAT3 is identified on the transcription factor layer at all-time points. MAPK1 is identified 4–8 h after EGF stimulation. PRKAR2B is identified later on (18–24 h after stimulation) on the protein layer. VAV1 is identified as a Steiner node in the static consensus graph 24 h after stimulation.

Additionally, we wanted to test in how far our integratory pathway-based approach is able to trace pathway crosstalk in the given data sets. In order to do so we chose a crosstalk mechanism which we expected to be reflected in the data set as it is not exclusively based on phosphorylation or ubiquitylation events. This mechanism is characterized by the activation of metalloproteinases (MMPs) by G-protein-coupled-receptors (GPCRs; Yarden and Sliwkowski, 2001). Upon activation MMPs cleave membrane-tethered ErbB ligands, which enables their binding to ErbB receptors, thereby positively regulating the ErbB signaling pathway. With EGFR being a receptor of the ErbB family our approach could identify a considerable number of the mentioned regulatory molecules in the consensus molecules (Table 2). Expression of different MMPs is observed starting at time point 4 h after EGF stimulation. Differentially expressed ErbB ligands for the different time points after EGF stimulation could be identified (such as self-induced EGF and AREG).

**Table 2:** **Consensus analysis**.

**Time after EGF stimulation [h]**	**1**	**4**	**8**	**13**	**18**	**24**
MMPs	−	MMP1	MMP1	MMP1	MMP1	MMP1
			MMP1	MMP1	MMP1	MMP2
			MMP1	MMP1	MMP1	MMP10
ErbB ligands	−	−	−	EGF	AREG	AREG
					EGF	EGF

*Regulatory molecules identified on the gene layer that are hypothesized to be involved in the signaling crosstalk via GPCRs and MMPs. GPCRs activate MMPs which then cleave the membrane-bound ErbB ligands leading to activated ErbB signaling (Yarden and Sliwkowski, 2001). Although differential expression is not direct evidence for the activity of these molecules, such regulatory mechanism can be hypothesized here*.

### Exploiting dynamic information of coupled time course data sets

**Figure 6:**
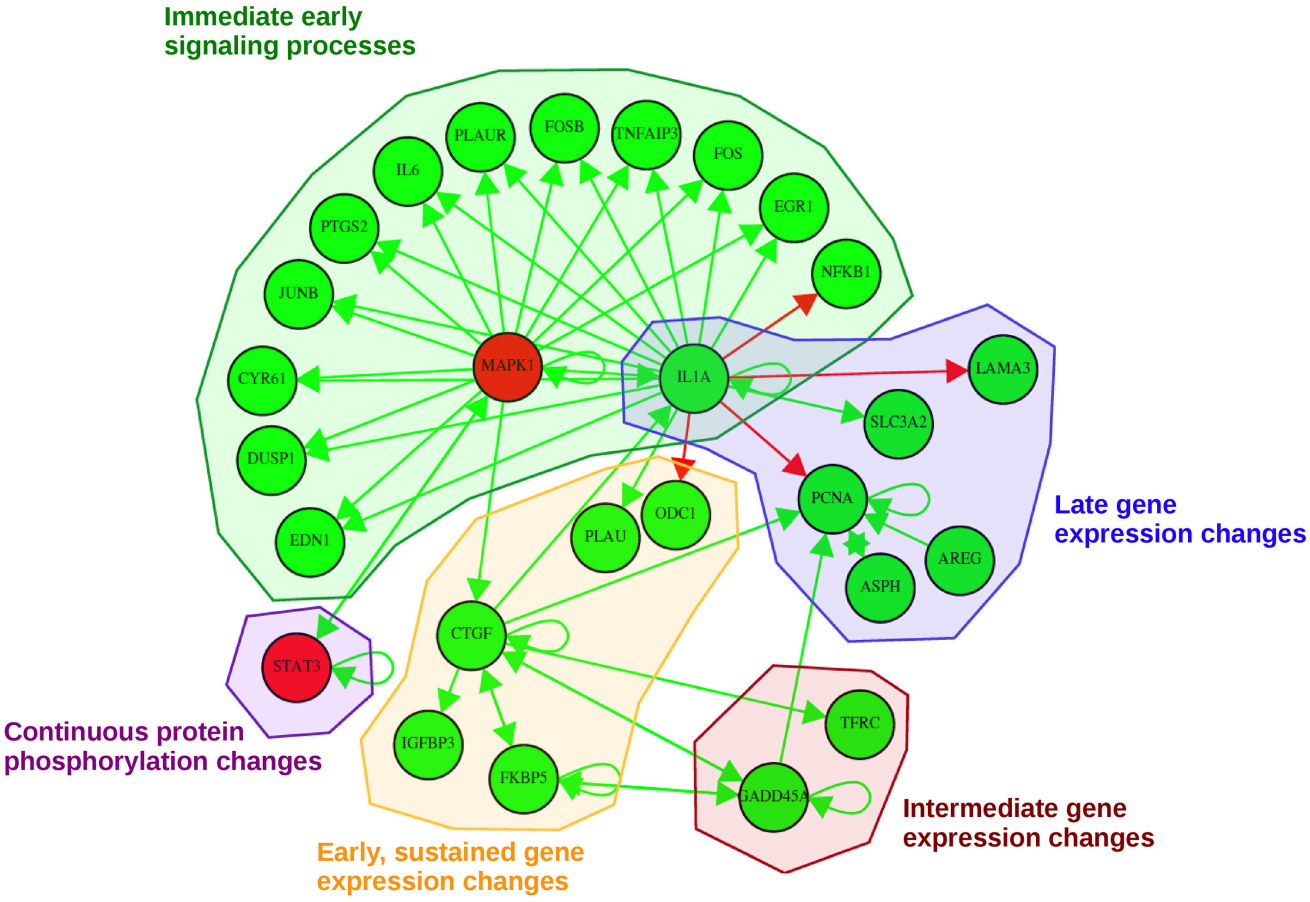
**Probabilistic network displaying result of the consensus-based dynamic analysis**. For network inference all consensus genes and proteins at any time point were considered. For visualization of the dynamic bayesian network a probability threshold was chosen corresponding to a probability of an edge to be present by chance of 0.15. Five groups could be identified comprising direct immediate early signaling processes, continuous protein phosphorylation changes, late gene expression changes, intermediate gene expression changes and early, but sustained gene expression changes upon stimulation. Activating regulatory effects are depicted with green edges whereas inhibiting regulatory effects are depicted as red edges. Consensus protein nodes are colored in red, consensus transcript nodes in green. Activation/inhibition refers to changes in the regulatory effects initiated by EGF stimulation, not to activated or inhibited expression.

Our pathway-based approach additionally enables the utilization of the complete time-series for each molecule in order to generate a probabilistic network displaying those nodes of the network with a high posterior probability of interaction. The dynamic analysis is based on the simplifying assumption of a gradual change in signaling over time, as existing high-frequency components are not considered due to the small sampling rate. Each consensus molecule at any time point after EGF stimulation was taken into account. With this approach we obtained the probabilistic network displayed in Figure 6. This network is a reduced way to look at activating or inhibiting relationships between consensus proteins and genes. Here, we observe mainly activating relationships corresponding to an activation of the regulatory effect of EGF stimulation and not to upregulation directly. Likewise an inhibiting relationship in the network does not imply a downregulation, but the inhibition of the effects induced by EGF stimulation.

In total, we could identify five subgroups in the consensus-based dynamic network by mapping them to the times in which they are part of the consensus graphs (Figure 6): (1) immediate early signaling processes, (2) early, but sustained gene expression changes, (3) intermediate gene expression changes, (4) late gene expression changes, and (5) continuous protein phosphorylation changes. In the group of the “immediate early signaling processes” most early response genes that were identified in the static consensus profiles are activated by the protein MAPK1 and the gene *IL1A*. This group reflects early phosphorylation induced transcriptional changes. The next group, consisting of five genes, is the group of “early, but sustained gene expression changes” upon EGF stimulation. It includes *CTGF*, a connective growth tissue factor. Its regulation is activated by MAPK1, *FKBP5, GADD45A* and also self-activation is observed. *CTGF* itself has activatory influence on gene members of its own group (*IGFBP3, FKBP5*), but also on members of the “intermediate gene expression changes” group and the “late gene expression changes” group. Two further members (*PLAU* and *ODC1*) are influenced by *IL1A*, a hub gene in the network, which we assigned to the “immediate early signaling processes” group and to the “late gene expression changes” group, as it shows immediate membership in the static consensus graphs, but also a late response profile. A small group showing intermediate gene expression changes comprises *TFRC* and *GADD45A*. We observe in the graph that *GADD45A* activates itself, but also *PCNA*, a gene of the “late gene expression changes” group. *PCNA* is additionally self-activated, as well as externally activated by the ErbB ligand *AREG* and *ASPH*, the aspartate beta-hydroxylase. *AREG* and *ASPH* are upregulated late after EGF stimulation. *IL1A* also activates *SLC3A2*, the solute carrier family 3 member 2, and inhibits *LAMA3*, a proliferating cell nuclear antigen, laminin alpha 3. The second protein being part of the network is the transcription factor STAT3. The changes in STAT3 phosphorylation are found in the consensus graphs over all time points, thus we assign it to the group of “continuous protein phosphorylation changes.” Beside the activating influence of MAPK1 also autoregulation of STAT3 can be detected.

### Time profile clustering

**Figure 7:**
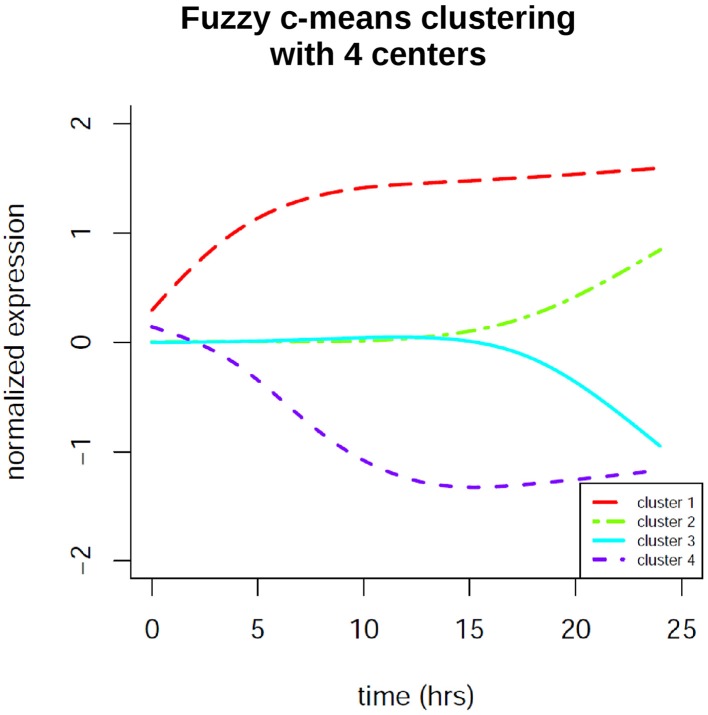
**Fuzzy c-means time profile clustering revealed 4 co-regulation clusters with distinct cluster sizes**. Two of the clusters exhibit positive regulation and two exhibit negative regulation. Both positive and negative clusters each comprise one cluster of immediate regulation and one of delayed regulation. The clusters contain both protein and gene expression changes. Cluster membership is listed in the Supplementary Material.

In order to identify co-regulation patterns in the signaling response after EGF stimulation we performed time profile clustering. We obtained four dynamic co-regulation patterns of which two exhibit positive regulation and two exhibit negative regulation. Both positive and negative clusters each comprise one cluster of immediate regulation and one of delayed regulation. The clusters are depicted in Figure 7. Corresponding molecule membership in the four different clusters is listed in the Supplementary Material (Table S1). Cluster 1 is immediately activated and thus contains various immediate early genes, but also the proteins MAPK1 and STAT3, which are part of the consensus-based dynamic analysis. Compared to the groups identified in the latter analysis this cluster constitutes the immediate early signaling processes together with early, but sustained gene expression changes. Cluster 2 is the biggest cluster with 52 members and is the delayed positively regulated cluster. Cluster 3 only comprises two members (RARRES3 and SLC3A2), both of which are showing a delayed negative dynamic co-regulation. Cluster 4 is the early negatively regulated cluster.

### Time course integration

**Figure 8:**
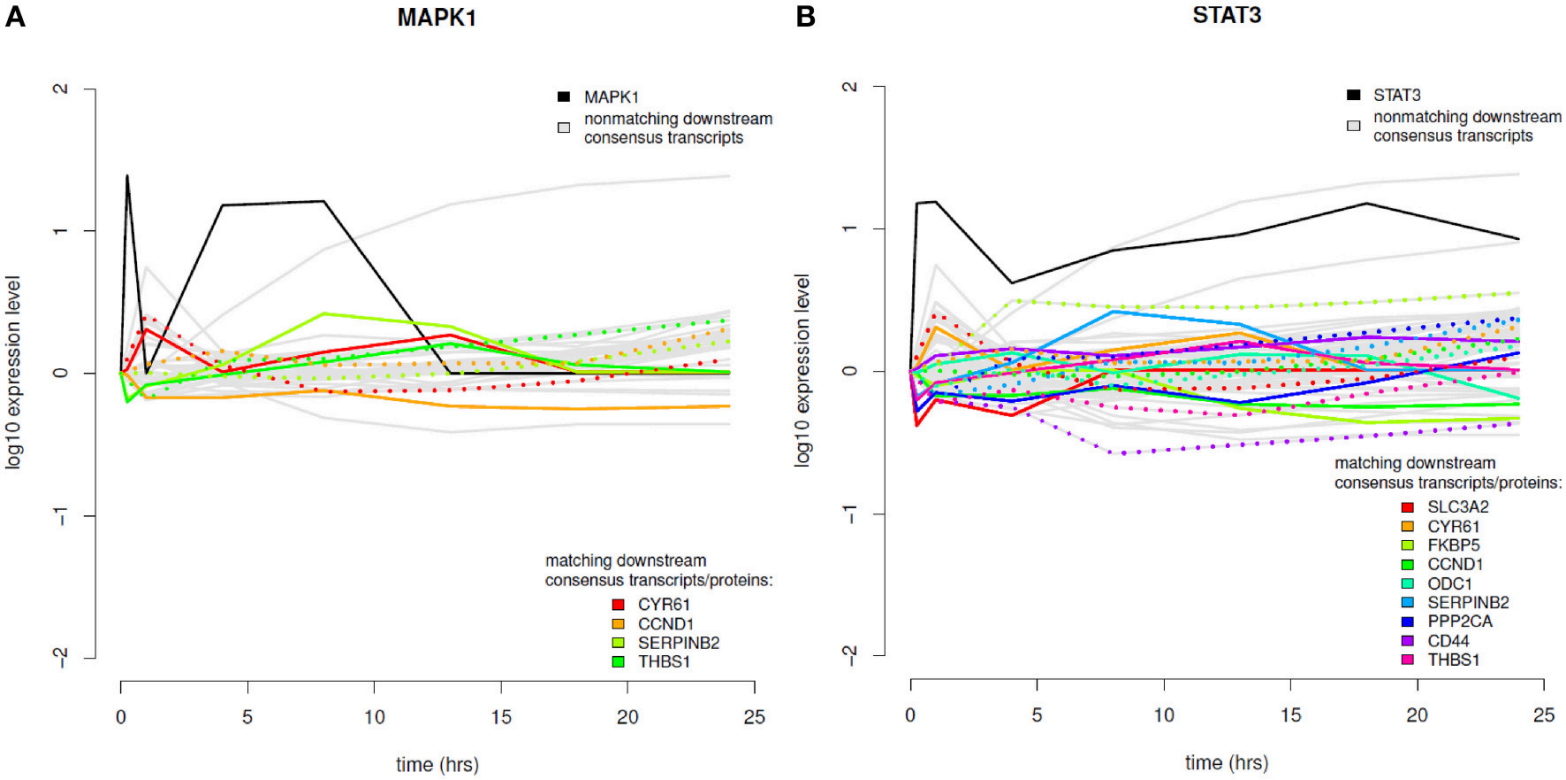
**Time course integration. (A)** MAPK1 downstream consensus transcripts identified were mapped to differentially abundant proteins. **(B)** Time course integration for downstream consensus transcripts of STAT3. Note that the measurement range of the expression profiles across platforms can vary. Phosphoprotein time course data is shown in solid, black lines, non-matching transcript data in solid, gray lines and matching transcript and proteome data in rainbow color palette with proteins depicted as solid lines and transcripts depicted as dotted lines.

The results of the time-course integration based on the consensus analysis results are displayed in Figure 8 and in the Supplementary Material (Figure S8). Of the five phosphoproteins that were measured over time in the coupled data set we could identify four phosphoproteins with their downstream transcripts being part of our consensus analysis and mapping to differentially abundant proteins (MAPK1, STAT3, MAPK14, and PRKAR2B). MAPK1 downstream analysis revealed four transcripts (Figure 8A), which mapped to significantly differential proteins, CYR61—cysteine-rich angiogenic inducer 61, CCND1—cyclin D1, SERPINB2—serpin peptidase inhibitor, clade B, member 2, and THBS1—thrombospondin 1. MAPK1 itself shows increased phosphorylation levels in the very beginning after EGF stimulation and again between 1 and 13 h after EGF stimulation. In regard to temporal coordination CYR61 shows correlating temporal expression on the transcript and protein layer up to time point 4 h after EGF stimulation, but then a rather opposed pattern. CCND1 belongs to the group of cyclins and thus exhibits a specific expression and degradation pattern over the cell cycle, in this way contributing to the temporal coordination of mitotic events. Here we can observe an opposed temporal pattern of transcripts and proteins over the whole timespan measured: While on the mRNA layer, CCND1 shows higher expression levels after EGF stimulation, the corresponding proteins are found at lower levels over the whole time course. High mRNA-to-protein levels have already been reported by Waters et al. (2012). In the time-course SERPINB2 shows slowly rising levels of transcripts after EGF stimulation, whereas on the protein layer there is a direct decrease, an intermediate increase, and a second decrease again to the 0-level at 18 h after EGF stimulation. THBS1 protein levels are similar to that of SERPINB2, however, here we observe rather correlating transcript levels in the beginning and deviating ones after the 18 h time point.

STAT3 is the phosphoprotein showing the most downstream transcripts that match to significantly regulated proteins (Figure 8B). STAT3 itself shows sustained high expression levels over the whole time-course. All MAPK1 downstream transcripts that are part of the consensus analysis also belong to the downstream transcripts of STAT3. Further ones are *SLC3A2, FKBP5, PPP2CA, CD44*, and *ODC1*. All of these except for *ODC1* show anti-correlating patterns between transcripts and proteins until 4 h after EGF stimulation. For later time points most pairs exhibit correlating behavior. MAPK14 also has *CYR61, CCND1*, and *SERPINB2* as downstream targets with corresponding proteins being significantly differentially abundant, whereas for PRKAR2B only *CYR61* could be identified.

## Discussion

### Pathway layer based integration

In the downstream and upstream analyses the results indicate that pathway identification based on differentially abundant phosphoproteins and differentially expressed transcripts is effective. In both pathway sets those pathways known to be activated by EGF stimulation were identified reliably in the different databases, expectedly the “EGF signaling pathway” itself. This shows, that the two data sets are in concordance on the pathway layer even if they are measured on different cellular layers and analyzed individually. Based on these initial results a pathway-based integration was considered to be constructive. However, downstream and upstream analyses might also introduce false positive findings, which we aimed to reduce from further analysis steps by the subsequent intersection analysis. The small set of phosphoproteins measured over time gives a strong basis for the pathway layer based integration as they were selected carefully for the experiment and belong to key pathways in EGF signaling. However, a larger set of phosphoprotein data as obtained now e.g., from mass-spectrometry approaches could lead to more robust results.

### Consensus analysis enables identification of regulatory dynamics

In order to evaluate our methods it is important to first classify the data according to their temporal transcriptional domains. According to Avraham and Yarden (2011) feedback mechanisms in EGFR signaling can be assigned to two temporal domains, one of them being the immediate group which includes receptor endocytosis, secondary phosphorylation and further protein modifications, the other constituting the late group which includes newly synthesized adaptors, transcriptional repressors, RNA-binding proteins and phosphatases of the mitogen-activated protein kinase (MAPK) pathway. Especially the integrated data with parallel time points between 1 and 24 h after EGF stimulation thus reflects the late group capturing the transcriptional regulation with a wave-like regulation of immediate early genes (IEGs), delayed early genes (DEGs), secondary response genes (SRGs; Avraham and Yarden, 2011) and their corresponding subsequent protein expression. IEGs are known to induce transcriptional changes of DEGs which then reduce the regulation of IEGs in a feedback subsequently, but initiate regulation of SRG expression. Based on this transcriptional regulation scheme the measured time points in the investigated data sets capture stimulation of both IEGs and DEGs 1 h after EGF stimulation while in subsequent time points we expect only regulation of SRGs, conferring the stable cellular phenotype.

We used the static consensus analysis in order to generate a static view on the integrated networks at each time point. Via static consensus profiles we can identify transcription factors with regulatory effects and their regulated consensus molecules on the gene layer at the 1 h time point. A large number of those genes were already reported to be IEGs in the cellular response to growth factor stimulation according to Tullai et al. (2007). PLAU and CTGF, regulated as well at later time points, apparently have an additional function in the definition of the phenotype. The two-phase regulation pattern indicates 2-fold tasks and can be interpreted to underly direct or indirect auto-feedback regulation.

The static consensus profiles of most SRGs, in contrast, are supposed to show a sustained activity. This is exactly what we find in our consensus graph analysis.

Due to the low number of differentially abundant phosphoproteins as a starting point the number of intersecting proteins from downstream and upstream analyses are low, as well. MAPK1 is involved in a variety of cellular growth processes such as proliferation and differentiation, thus its presence in the consensus graph corresponds well to the expected cellular response after EGF stimulation. As a regulatory subunit of the cAMP-dependent protein kinases PRKAR2B is involved in various cellular functions. With its late activity we suspect an involvement in the cellular reconstruction processes taking place for the final phenotype definition. The VAV proteins are guanine nucleotide exchange factors that activate pathways leading to cytoskeletal actin rearrangements and transcriptional alterations (Han et al., 1998). Thus, its functional association can be linked to cellular restructuring during proliferation.

In EGF signaling several pathways are involved which do not only process signals in a linear way but also enable cross-pathway regulatory influence on transcription. Oda et al. (2005) tried to compress all known signaling interactions into a comprehensive pathway map, resulting in a bow-tie architecture signaling pathway. As this network has to convey fine-tuned messages, it is deducable that slight dysregulation results in pathological transcriptional responses. Many crosstalk mechanisms have been investigated in more detail, most of them under pathological conditions. However, in order to understand the consequences of such dysregulation it is essential to also have a detailed understanding of physiological pathway crosstalk mechanisms. This is why we reviewed the consensus molecules in terms of their possible role in the crosstalk described by Yarden and Sliwkowski (2001). The large number of identified consensus molecules implicated in this crosstalk on the gene layer supports our hypothesis, that they are part of this signaling crosstalk mechanism.

As the described regulatory dynamic patterns are based on two independent data sets from different platforms we suppose that this pattern is not identified due to measurement bias and thus has a biologically relevant function in the cellular response.

### Identification of regulatory mechanisms by exploiting dynamic information of coupled time course data sets

In order to fully exploit the dynamic information of the time course data sets, we inferred a probabilistic network based on all consensus molecules. This network enables an identification of important players in the cellular response to EGF as well as the determination of inhibitory or activating regulation patterns.

The consensus proteins which are part of the dynamic network are MAPK1 and STAT3, both being part of the starting phosphoprotein data set. This indicates, that their important role in EGF signaling can be confirmed as such via the transcriptomic data set. STAT3 is a transcription factor, which is phosphorylated upon growth factor stimulation of the cell and builds homo- or heterodimers, which can then translocate to the nucleus and activate transcription (Park et al., 1996). It has multiple target genes with its protein products being involved in proliferative processes. MAPK1 is associated with cellular processes such as proliferation, differentiation and transcriptional regulation. Both show a self-activation as well as a mutual activation, which illustrates their functional relevance in EGF signaling. This regulatory interaction between MAPK1, also known as ERK2, and STAT3 is triggered via the activation of the MAPK/ERK cascade upon EGF stimulation, leading to MAPK1 phosphorylation by upstream kinases. STAT3 transcriptional activation by phosphorylation of STAT3 pS727 is then performed by the serine/threonine kinase ERK (Zhang and Liu, 2002), leading to activation of STAT3, which then acts as transcription factor and initiates the expression of downstream target genes. Target genes of STAT3 that might lead to further activation of MAPK1 are e.g., downstream transcription factors, multiplying indirectly the effective activation, or EGFR allowing for binding of more EGF. Furthermore, JAK2 is a target gene of STAT3, which can contribute to positive auto-feedback of STAT3 via the JAK-STAT pathway (Dauer et al., 2005).

Beside the already discussed early regulation processes and the protein phosphorylation changes of STAT3, the other identified groups are particularly interesting for further interpretation: The regulation of *CTGF*, the connective growth tissue factor, is activated by MAPK1, *FKBP5, GADD45A* and by itself. Interestingly, we observe auto-feedback regulation here, as already suspected from the static consensus profiles. *CTGF* is a hub gene in the consensus-based dynamic network, so the activation of its downregulation upon EGF stimulation is associated with downregulation of other genes in this cluster, such as *FKBP5*, or genes of the “intermediate gene expression changes” group. One of these is *GADD45A*, the growth arrest and DNA-damage-inducible alpha, which activates the regulation of PCNA. It is known to comprise increased transcript levels when cells are subjected to arrest conditions, treatment with DNA-damaging agents and environmental stresses (Hollander et al., 1993), thus we suspect the experimental design of the experiment with the chosen growth arrest time to be of no direct harm to the cells. PCNA, the proliferating cell nuclear antigen, is a cofactor of DNA polymerase delta and plays a central role during DNA replication. In DNA damage response it is positioned at the replication fork coordinating replication with DNA repair and DNA damage tolerance pathways (Cazzalini et al., 2014). Thus, its function is intensely needed in the phase of cellular remodeling and proliferation. The link between *GADD45A* and *PCNA*, that we determined with our integrative analysis, was previously reported (Chen et al., 1995).

*AREG* is upregulated in the “late gene expression changes” group as part of the regulatory pathway crosstalk loop via metalloproteinases described above and presumably provides an additional amplifying cellular way of an activation cascade after initial EGF stimulation. Also ASPH, which is thought to play an important role in calcium homeostasis (Treves et al., 2000), is part of this group. With its diverse roles e.g., as a messenger between cellular compartments calcium regulation is essential for proliferating cells.

*IL1A*, as another hub in the network, has immediate and late regulatory influence. In the “late gene expression changes” group it activates *SLC3A2*, solute carrier family 3 member 2, and inhibits *LAMA3*, proliferating cell nuclear antigen, laminin alpha 3. With their functions in regulating intracellular calcium levels, amino acid transport, formation and function of the basement membrane, cell migration and mechanical signal transduction and DNA replication, this part of the network rather shows the expression changes which represent the secondary (late) response of the cells.

In summary, we identified MAPK1, *IL1A* and *CTGF* as main players driving EGF stimulation response in the cell. Interestingly, we could detect the link between *GADD45A* and *PCNA* in two independent high-throughput time course data sets measured on different platforms using our pathway-based integration approach. As a matter of course, with a higher temporal resolution of the coupled time course measurements more accurate results can be identified by our approach, as less intermediate time points need to be estimated. To gain insight into the biological response after an external stimulation at least four time points after the stimulation time point are necessary, though there is a high information content in such coupled data sets on the different cellular layers. The chosen time points and the temporal resolution, however, need to be adjusted specifically to the cellular signaling dynamics and the stimulation of choice in order to reflect the crucial time points of regulation.

### Time profile clustering identifies four dynamic co-regulation patterns ruling EGF signaling

With our time profile clustering approach we could identify four co-regulation patterns with distinct functions in the cellular response to EGF signaling. Cluster 1 contains many of the directly upregulated immediate early genes. Most of these are in fact downregulated again after their early response, which is not reflected by this cluster, as it contains also a considerable number of genes that are secondary response genes and are only upregulated at later time points (such as MMP1 or MMP10) or immediate early genes which are upregulated again at later time points (PLAU or IL1A). Our hypothesis, that cluster 2 includes mainly genes upregulated as secondary response genes, responsible for the phenotype definition, holds true, when having a closer look to the members: We observe *CCND1*, the cyclin family protein, *ANXA1* and *ASPH, LAMA3* and *AREG*, which were identified in the consensus-based dynamic analysis in the group of late gene expression changes, *VEGFC*, a vascular endothelial growth factor promoting angiogenesis, *CCND2*—cyclin D2, *NME1*—nucleoside diphosphate kinase 1, which has been associated with high tumor metastatic potential based on different studies (MacDonald et al., 1996) and many more genes which act during cellular proliferation and migration. As cell cycle inhibitory protein coding genes we can observe the membership of *CDKN1A*, the cyclin-dependent kinase inhibitor 1A, which is tightly controlled by transcription factor p53 (He et al., 2005). Its membership in cluster 2 might be due to the high importance of balancing proliferation processes against growth stimulating processes in physiological tissue. Further we observe *PTHLH*, the parathyroid hormone-like hormone, to be part of this cluster, which regulates the epithelial-mesenchymal interactions during formation of mammary glands and teeth (Wysolmerski, 2012). Additionally the protein PRKAR2B is part of this cluster, indicating its late activation, which we already observe in the phosphoproteome data individually. However, here we see the confirmation that it is part of the consensus data from the two independent data sets generated on different platforms. Also *MMP2* is part of cluster 2 as well its regulatory counterpart, *TIMP1*, a metallopeptidase inhibitor. As the other metalloproteinases identified in the static consensus graphs (*MMP1* and *MMP10*) are not members of cluster 2, but of the immediately positively regulated cluster 1, it can be assumed, that *TIMP1* activation might also have a negative regulatory impact on these late after EGF stimulation. In the delayed downregulated cluster 3 we observe RARRES3, the retinoic acid receptor responder 3, which is known for its growth inhibitory effects (Hsu and Chang, 2015). A late downregulation thus can have the function of preventing contrasting growth signals. SLC3A2, the solute carrier family 3 member 2, encodes a subunit of a cell surface transmembrane protein complex responsible for regulation of L-type amino acid transport, which is essential for cellular growth and proliferation (Yanagida et al., 2001). Cluster 4, the early negatively regulated cluster, comprises *CTGF*, the connective tissue growth factor, whose downregulation might enhance proliferation of cells upon EGF stimulation. A further member is *IGFBP3*, the insulin-like growth factor binding protein 3, which potentiates insulin-like growth factor action and thereby also stimulates growth promoting effects (Cubbage et al., 1990). Supposedly, the cells do need less proliferating activation via IGF, when there is the growth-promoting stimulation of EGF. This underlines again that signaling patterns are tightly regulated in regard to their dynamics.

### Time course integration of consensus graphs with proteome data

We were interested in how far our approach reveals the dynamics of elements in the regulatory cascade of a stimulation induced phosphorylation cascade triggering a specific gene expression, which then leads to the generation of proteins needed in the cellular response to that particular stimulation. Therefore, after integrating the phosphoproteome data in the first pathway layer based integration, we integrated in a second step also the proteome data with the results of our pathway-based integrative analysis dynamically. The delay between consensus transcript generation and their corresponding protein generation reflects the time the cell needs for the complete translational and post-translational process. However, it is known that differences in protein abundance are only attributable to mRNA levels by about 20–40% (Brockmann et al., 2007). This underlines the importance of post-translational modification and is the reason why we assumed the correlation between increasing and decreasing transcript expression and corresponding protein generation to be rather marginal.

For the interpretation of these results we need to be aware of the different ranges of the expression ratios in the data sets of different platforms. Thus, a direct comparison of the expression levels between transcripts and proteins is not possible, however, a dynamic interpretation is feasible.

Dynamically, we observe both correlating and non-correlating expression level patterns between transcripts and corresponding proteins. Based on the time resolution of the measurements we assume the time delay reflecting the translational and post-translational processes to be not necessarily observable in the data, as they can lie in a wide time range. Indeed, correlating behavior seems not to be shifted in time in our analysis for certain transcripts (e.g., for CYR61 up to 4 h after EGF stimulation or THBS1 up to 13 h after EGF stimulation), however, when performed on a time-series data set with higher resolution, such time shifts might be observable. Non-correlating expression level patterns indicate post-translational modifications or a possibly very rapid degradation of mRNA or the protein product, which is not captured in the low resolution time measurements. Of the identified pairs CYR61 is a growth factor inducible protein which promotes the adhesion of endothelial cells (Brigstock, 2002), CCND1 is a protein contributing to coordination of mitosis. High levels of SERPINB2 have been observed to exhibit an anti-proliferative effect (Croucher et al., 2008). In the time courses we see an intermediate increase of its protein levels, but an overall anti-correlating pattern between protein and transcript levels. THBS1, thrombospondin 1, is known as angiogenesis regulator (Chandrasekaran et al., 2000). Its protein levels are similar to that of SERPINB2, however, here we observe rather correlating expression levels, indicating less post-transcriptional modification. Also changes in the correlation behavior can be observed, indicative for a secondary regulatory influence. This could be induced by variations in mRNA degradation, protein degradation rates or post-translational modifications.

From the transcript/protein pairs that are observed as part of the regulatory loops CYR61, THBS1, and CCND1 clearly have a high influence on EGF stimulated cells during cellular proliferation, differentiation and survival, while the detection of SERPINB2 is more intriguing. It is known to inhibit urokinase plasminogen activators (PLAUs), but its physiological function has not been characterized comprehensively, although activity in the adaptive immune response has been reported (Schroder et al., 2011). As we based the time-course integration on the consensus analysis the discussed time-courses are supported by both transcriptome and proteome data set. Thus, we hypothesize the interaction of SERPINB2 and PLAU, its inhibition target, to be of high relevance for proliferative processes. Our hypothesis is supported also by literature in the context of cancer: SERPINB2 has been associated with increased survival in breast cancer patients (Duffy, 2004).

With the integrated time-courses of phosphoproteins, downstream consensus-graph transcripts and their corresponding proteins the data implies an extensive post-translational modification of a number of proteins. This we see in the transcript/protein pairs investigated in detail here, but also in the downstream transcripts depicted in gray in Figure 8, with no corresponding proteins in the list of significantly differentially abundant proteins. Therefore, our results correspond to what is known about the low percentage of protein concentration variations that are affected by mRNA abundances directly (Vogel and Marcotte, 2012). However, our approach not only enables a general overall classification of correlating or anti-correlating transcript/protein pairs, but in addition a time-resolved interpretation of consensus-based regulatory processes.

### Comparison of separate data set analysis with integrated consensus-based analysis

To comprehensively assess the advantage of our data integration approach based on public pathway knowledge we compared its results with the ones gained by a separate analysis of the individual proteomic and transcriptomic data sets. Waters et al. (2012) performed a separate pathway analysis and reported network statistics, such as the number of nodes in the largest cluster, the number of edges in the network and the two primary hub nodes, however, this analysis was limited to data measured 0–4 h after EGF stimulation. Interestingly, the hub genes identified in the microarray based network were the transcription factors *FOS* and *EGR1*, while the hub proteins identified in the proteome data were EGFR and ITGB1. Comparing these results to our results from the pathway-based integrative analysis, we likewise observe FOS and EGR1 to be highly important regarding regulatory mechanisms during the initial cellular response. Yet, we additionally derived further information than what is given by the separate analysis: We evaluated these genes to play a significant role in the immediate early cellular reaction based on static consensus profiles. Furthermore, we saw that these are mainly influenced by *IL1A* and the phosphorylation of MAPK1 directly as well as indirectly. Based on the time profile clustering we saw on top that they belong to the early positively regulated cluster. The protein hubs that are identified via the separate analysis, however, cannot be found in our consensus analysis, as the consensus is confined to the small set of measured phosphoproteins.

In a second separate analysis of the proteomic and transcriptomic data sets Waters et al. (2012) performed separate gene set enrichment on the basis of differentially expressed proteins and transcripts. The three most significant biological processes identified for the transcriptomic data set were “cell cycle,” “mitosis,” and “protein folding,” while for the proteomic data set the most significant process was “protein synthesis.” In a comparison the authors found considerable differences in the gene set enrichment results. Although this type of analysis is widely used for gene expression data it is arguable in how far “gene set” and “protein set” enrichment should be compared directly due to the different biological layers the data and possibly also network knowledge originates from. Thus, we see an inherent problem in the simplified layer-unspecific comparison with subsequent interpretation. Additionally, the results allow no conclusions or hypothesis generation on the molecular level.

In summary, we conclude that the integrated analysis of the two data sets moves the focus to the dynamic interplay of regulatory mechanisms and enables a layer specific and detailed regulatory analysis of the cellular response to external stimulation.

### Comparison of data integration approaches in coupled high-throughput data sets

The data integration approaches applied by Waters et al. (2012) were based on RNA/protein pairs cross-referenced between the platforms. However, no layer-specific analysis was performed. In a canonical correlation analysis the 199 RNA/protein pairs comprising all measurement time points were investigated with the result of intense post-transcriptional regulation on the protein layer. The benefit compared to a simple correlation analysis is that it captures also concordance or disconcordance of pairs when a temporal delay is observed. With our time-course integration we could also observe this effect, individually for specific phosphoprotein initiated signaling cascades. With our approach it is additionally possible to analyze transcriptional and translational dynamics of each cascade individually.

In the integrative analysis of Waters et al. (2012) major cell processes of the combined data were then ranked to early (0–4 h), intermediate (8–13 h) and late (18–24 h) time domains after EGF stimulation. A general shift from categories “cytoskeletal organization” and “regulation of cell cycle” (0–4 h) toward anti-apoptotic and cell adhesion pathways (8–13 h) was observed. An increased representation of the “mitosis” category between 18 and 24 h after stimulation corresponded to an increase of mitotic cells monitored by flow cytometry in parallel. A direct comparison of the analyses results is not possible here, though the results we found in the consensus-based dynamic analysis of the data agree roughly with the results of Waters et al. (2012), when comparing the function of individual consensus molecules with the GO biological process category names. Although having category names enables in general a better overview of the data, it does not allow individual identification of regulatory interactions. Therefore, we consider our approach as valuable additional method in order to get a better understanding of the dynamic biological processes.

Furthermore, integrated signaling networks from all data sets were investigated in Waters et al. (2012). Not surprisingly, the microarray data set contributed the highest number of nodes in the merged network. Compared to the signaling networks from single data sets, the integrated network comprised increasingly linked nodes, reflected in the number of edges and the degree of the largest cluster reported. The two primary hub nodes of the integrated network were *FOS* and *SRC*, while the hub nodes in the network generated from exclusively microarray data were *FOS* and *EGR1*, generated exclusively from proteome data EGFR and ITGB1 and exclusively from phosphoproteome data STAT3 and MAPK1. Interestingly, we also found *FOS* and *EGR1*, as well as STAT3 and MAPK1 as consensus molecules in our consensus-based dynamic analysis with considerable regulatory influence during the cellular response after EGF stimulation. The proteome hub nodes EGFR and ITGB1, as well as the hub node *SRC* from the integrated network were not part of our results due to the low number of phosphoproteins measured in the study. However, we found already considerable amount of regulatory mechanisms when including only the phosphoproteome data set as initial data set in our analysis. The MMP cascades identified in the integrated analysis from Waters et al. (2012) as most robust response to EGF stimulation were identified as consensus molecule based process by our approach as well.

Unfortunately, in the integrated analysis of Waters et al. (2012) only time domains were considered in contrast to our individual time point analysis. This enables a rough summarized view on the signaling process, yet it does not fully exploit the information encoded in the dynamics. Likewise, the GO term analysis performed is based on a subset of RNA/protein pairs and results in a summarized interpretation, but it does not enable an individual regulatory mechanistic interpretation. Thus, we consider our approach as valuable complement in the analysis of coupled high-throughput data sets.

## Conclusion

The presented data integration approach shows a way to gain a much deeper understanding of biological processes if time-course measurements and data from different high-throughput platforms representing the different functional layers of the cell are combined. Our approach enables a functional linking of regulatory processes over the transcriptional and translational cycle, even if the temporal resolution of the example data set is quite low, data has only been measured on two functional cellular layers and the phosphoproteome data set is very limited. This sets the basis for the integration of further cellular layers, as following regulation upon external perturbation in a detailed way provides a much deeper understanding of biological processing.

Bioinformatic tools like the R package *pwOmics* promote the generation of coupled data sets as they offer the possibility of an integrated analysis and help to sort the vast data sets in a biologically interpretable manner. By applying the different analysis steps implemented in *pwOmics* we showed that biological interpretation is facilitated and the results correspond to current biological knowledge about EGF stimulation generated in low and high-throughput experiments. Furthermore, we identified interesting regulatory relationships that were not observed yet in physiological EGF signaling. As our approach considers data from the different functional cellular layers individually, it enables to identify the regulatory interplay between these layers. We have demonstrated this in the consensus analysis, which is able to identify the molecular response minutes to hours after stimulation as feedback mechanism with a wave-like regulatory pattern generated by IEGs, DEGs, and SRGs and their corresponding proteins. We could also identify previously published pathway crosstalk via activation of MMPs (Yarden and Sliwkowski, 2001). Furthermore, we could ascertain the link in EGF signaling between the two molecules GADD45A and PCNA, in the investigated data sets, which was previously reported (Chen et al., 1995). Interestingly, we also found PTHLH in the consensus molecules as part of the secondary cellular response, which is involved in the formation of mammary glands (Wysolmerski, 2012). Furthermore, we could identify the regulatory interaction of PLAU and SERPINB2 to be also of high relevance in physiological EGF signaling. Compared with the previously performed integrative analysis on the coupled data set we gain a complementary, and much more detailed view on cellular signaling processes, enabling the generation of biological hypothesis about individual regulatory mechanisms involved in the dynamic interplay of signaling pathways and feedback responses. With the examples stated above we could show, that our integrative approach is able to identify regulatory patterns, molecular interactions and dynamically orchestrated cellular response mechanisms.

In order to link the different functional cellular layers it is beneficial and necessary to integrate knowledge from public databases which builds a frame for placing and linking the individual analysis results. This has the advantage of utilizing a vast amount of collected and curated information, which stays unused otherwise and can add an additional information layer for interpretation of the data. On the other hand this prior knowledge also directs the results in a certain extent, thus the quality of the databases used has to be taken into consideration when interpreting the overall results. A further caveat is that the public database knowledge available in most databases is not cell type or tissue specific resulting in a generalized analysis. However, as more cell type or tissue specific knowledge is collected such databases can be build up and integrated in the presented analysis workflow.

In the consensus-based dynamic analysis we make the simplifying assumption of a gradual change of signaling over time. Clearly, this does not hold true for individual cells and still is a rough assumption for a set of cells as there have been found oscillatory mechanisms which work at high frequencies (Avraham and Yarden, 2011), for example, and which are purely not identifiable via such a time resolution. However, we can still gain a lot of knowledge about the regulatory processes that are encoded in the comparably slow dynamic processes. Of course, there can be even more biologically functional layers measured in high-throughput experiments in a parallel manner over time, such as siRNA, epigenetic influences etc. At the moment such data sets are still rare, but we expect them to be generated increasingly. It will be interesting for future projects to include such additional layers into an integrative analysis.

We showed that the hypotheses on regulatory mechanisms generated via our integrative approach could be confirmed with independent low-throughput data sets. Although such time-course data sets measured in parallel enable a detailed analysis, it is not yet possible to infer from these data sets every regulatory aspect in detail. Nevertheless, our approach is a step toward portraying the whole picture of regulatory influences on the molecular level.

## Availability

Main analysis steps of the pathway-based integration approach of coupled time-series omics data described in this manuscript are implemented in the R package *pwOmics* (Wachter and Beissbarth, 2015).

## Author contributions

AW developed the method, performed data analysis and wrote the manuscript. TB conceived the design, envisioned the project and revised the manuscript.

### Conflict of interest statement

The authors declare that the research was conducted in the absence of any commercial or financial relationships that could be construed as a potential conflict of interest.

## Footnotes

^1^www.quiagen.com/ingenuity.

^2^http://omics.pnl.gov.

^3^Morgan, M., Carlson, M., Tenenbaum, D., and Arora, S. *AnnotationHub: Client to Access AnnotationHub Resources*. R package version 2.0.0.

## Supplementary material

Figure S1 **Static consensus profiles of all members of the static consensus graphs**. Color coding corresponds to the one used in the static consensus graphs (red, consensus proteins; yellow, steiner node proteins; lightblue, consensus transcription factors; green, consensus genes).

Figure S2 **Static consensus graphs for time points 1 h after EGF stimulation**.

Figure S3 **Static consensus graphs for time points 4 h after EGF stimulation**.

Figure S4 **Static consensus graphs for time points 8 h after EGF stimulation**.

Figure S5 **Static consensus graphs for time points 13 h after EGF stimulation**.

Figure S6 **Static consensus graphs for time points 18 h after EGF stimulation**.

Figure S7 **Static consensus graphs for time points 24 h after EGF stimulation**.

Figure S8 **Time course integration for phosphoproteins MAPK14 and PRKAR2B**. Downstream consensus transcripts identified for MAPK14 and PRKAR2B were mapped to differentially abundant proteins. Note that the measurement range of the expression profiles across platforms can vary. Phosphoprotein time course data is shown in solid, black lines, non-matching transcript data in solid, gray lines and matching transcript and proteome data in rainbow color palette with proteins depicted as solid lines and transcripts depicted as dotted lines.

Table S1 **List of molecule cluster membership in the time profile analysis**. Data origin is encoded in the abbreviation after each protein/gene name (_g, microarray data; _p, proteome data).

Table S2 **Lists of pathways identified in the downstream analysis based on the phosphoprotein data for time points 0.25, 1, 4, 8, 13, 18, and 24 h after EGF stimulation**. Table includes information about the pathway database used for pathway identification (as part of their ID) and the corresponding pathway names.

Table S3 **Lists of pathways identified in the upstream analysis based on the differentially expressed transcripts for time points 1, 4, 8, 13, 18, and 24 h after EGF stimulation**.
