# Supplementary figures and images for: Decoding Cellular Dynamics in Epidermal Growth Factor Signaling Using a New Pathway-Based Integration Approach for Proteomics and Transcriptomics Data
Source: Front Genet. 2016 Jan 7;6:351. doi: 10.3389/fgene.2015.00351 (PMC4703778; doi:10.3389/fgene.2015.00351)

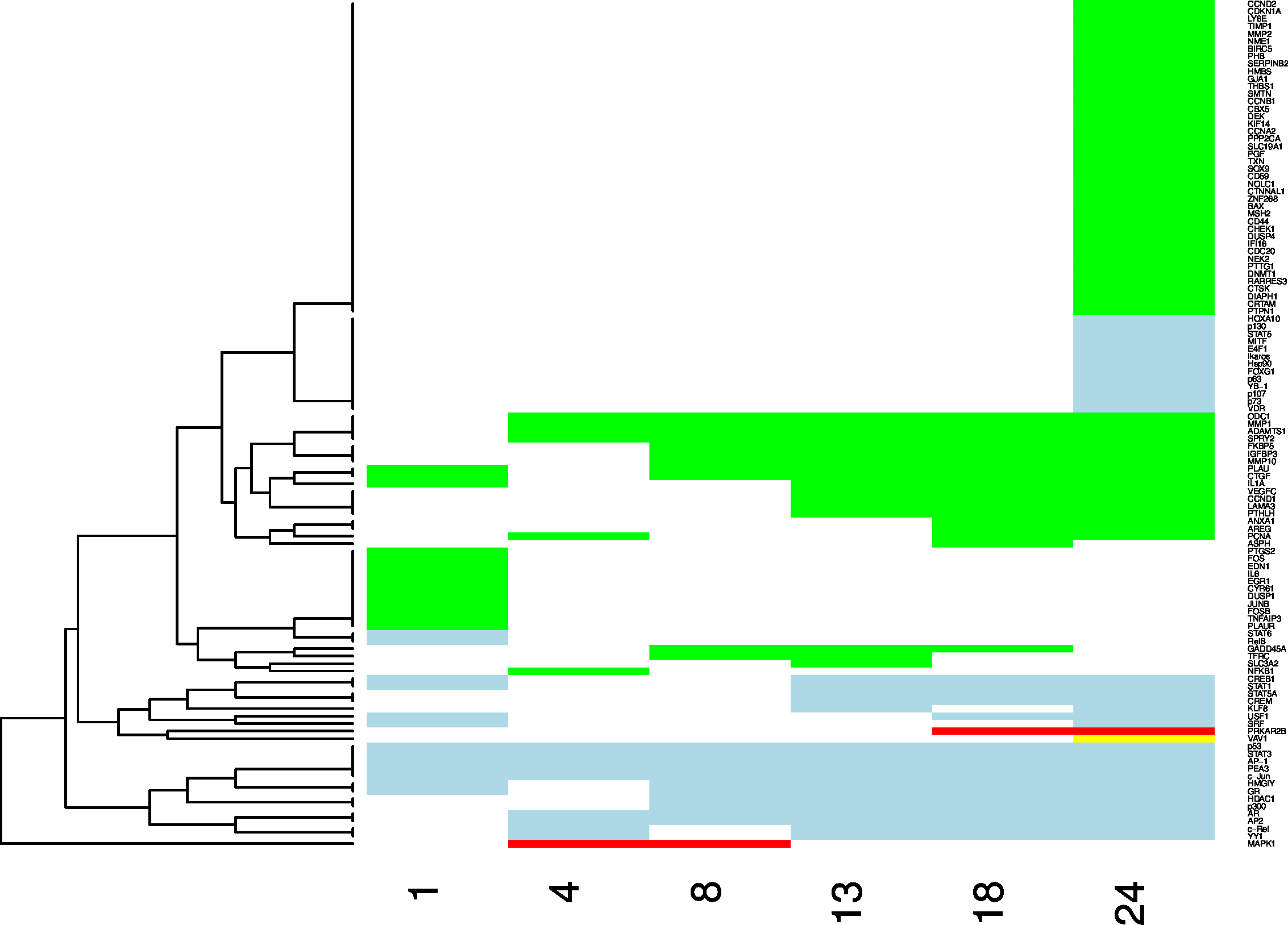

Supplement: Figure S1 — Static consensus profiles of all members of the static consensus graphs. Color coding corresponds to the one used in the static consensus graphs (red, consensus proteins; yellow, steiner node proteins; lightblue, consensus transcription factors; green, consensus genes). [file Image1.JPEG]

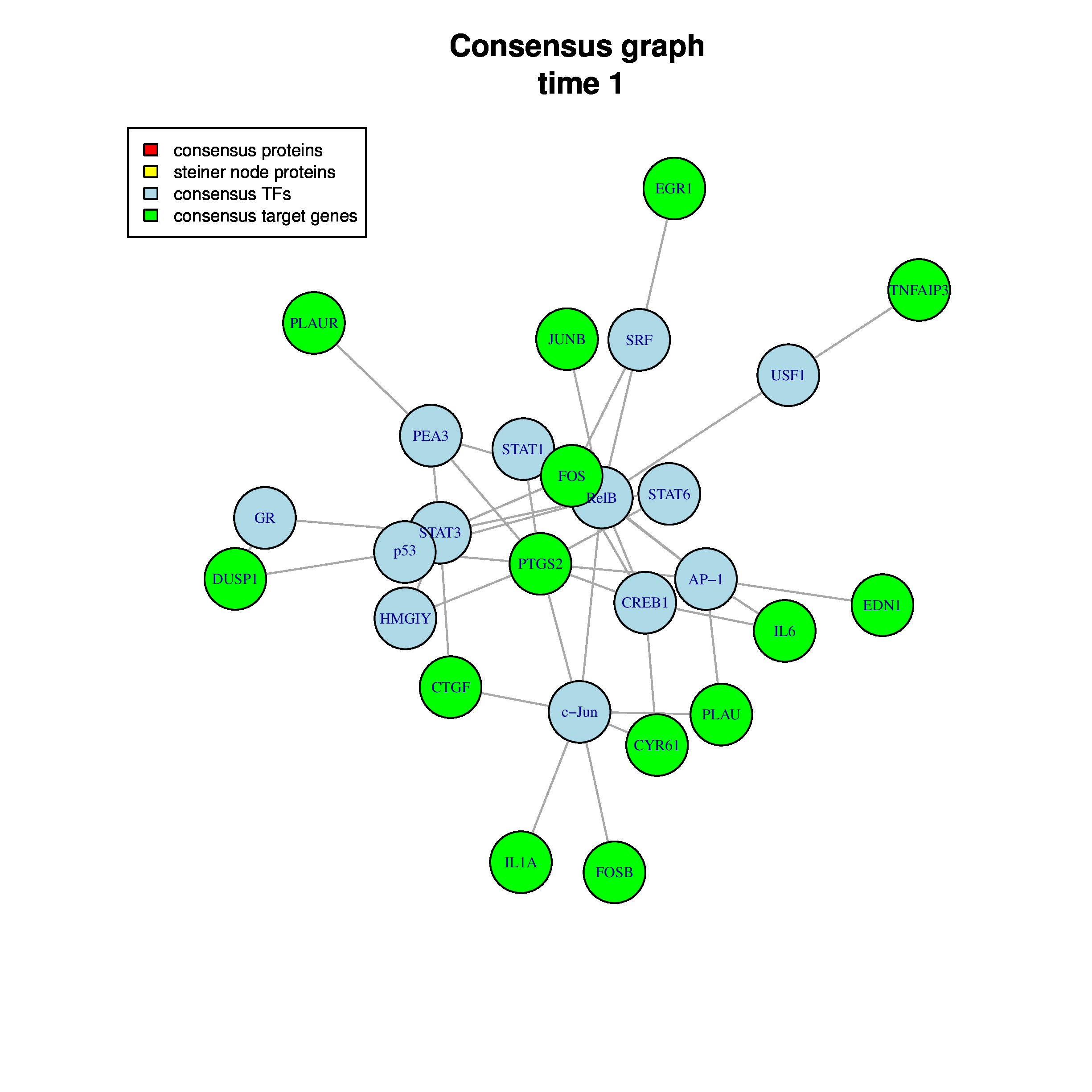

Supplement: Figure S2 — Static consensus graphs for time points 1 h after EGF stimulation. [file Image2.JPEG]

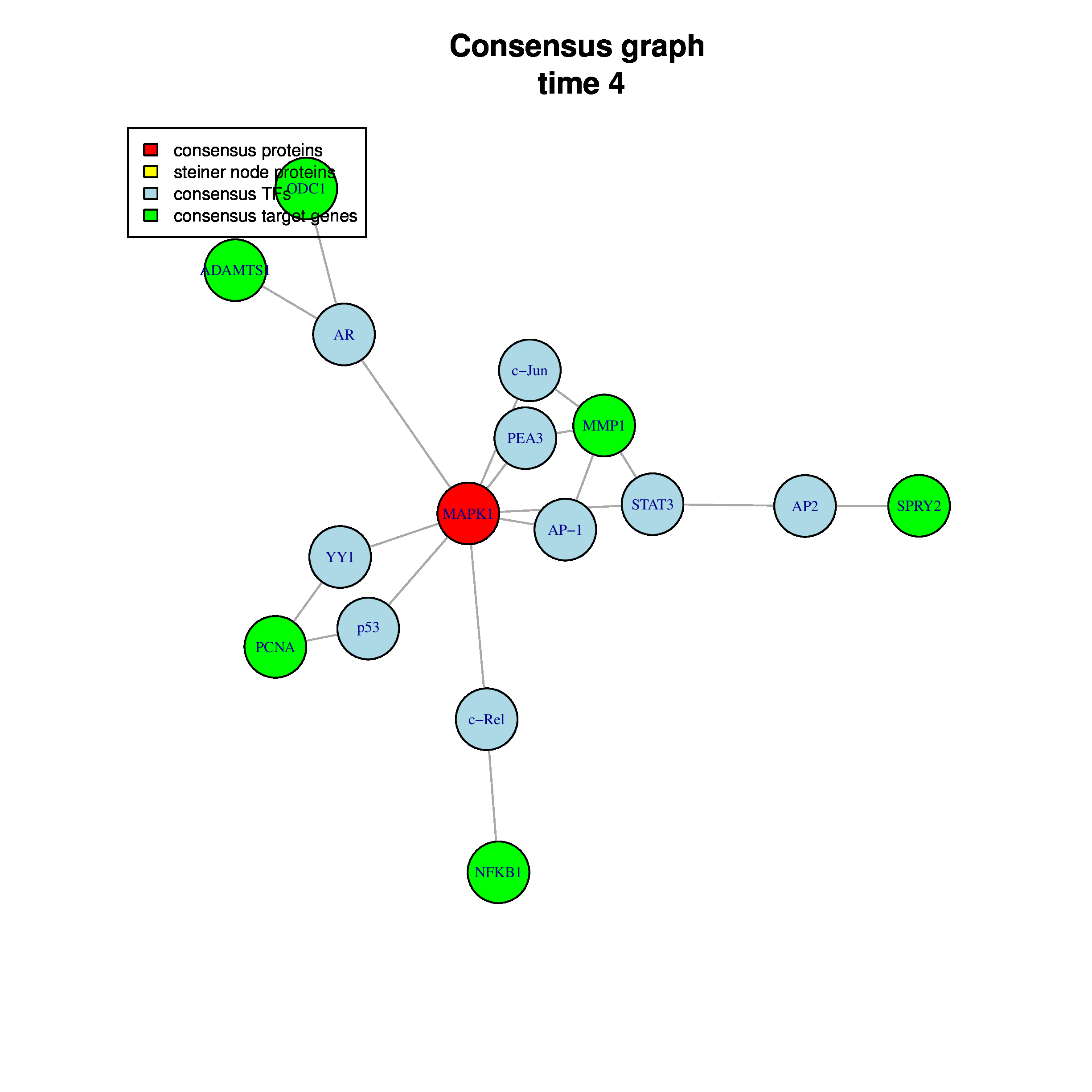

Supplement: Figure S3 — Static consensus graphs for time points 4 h after EGF stimulation. [file Image3.JPEG]

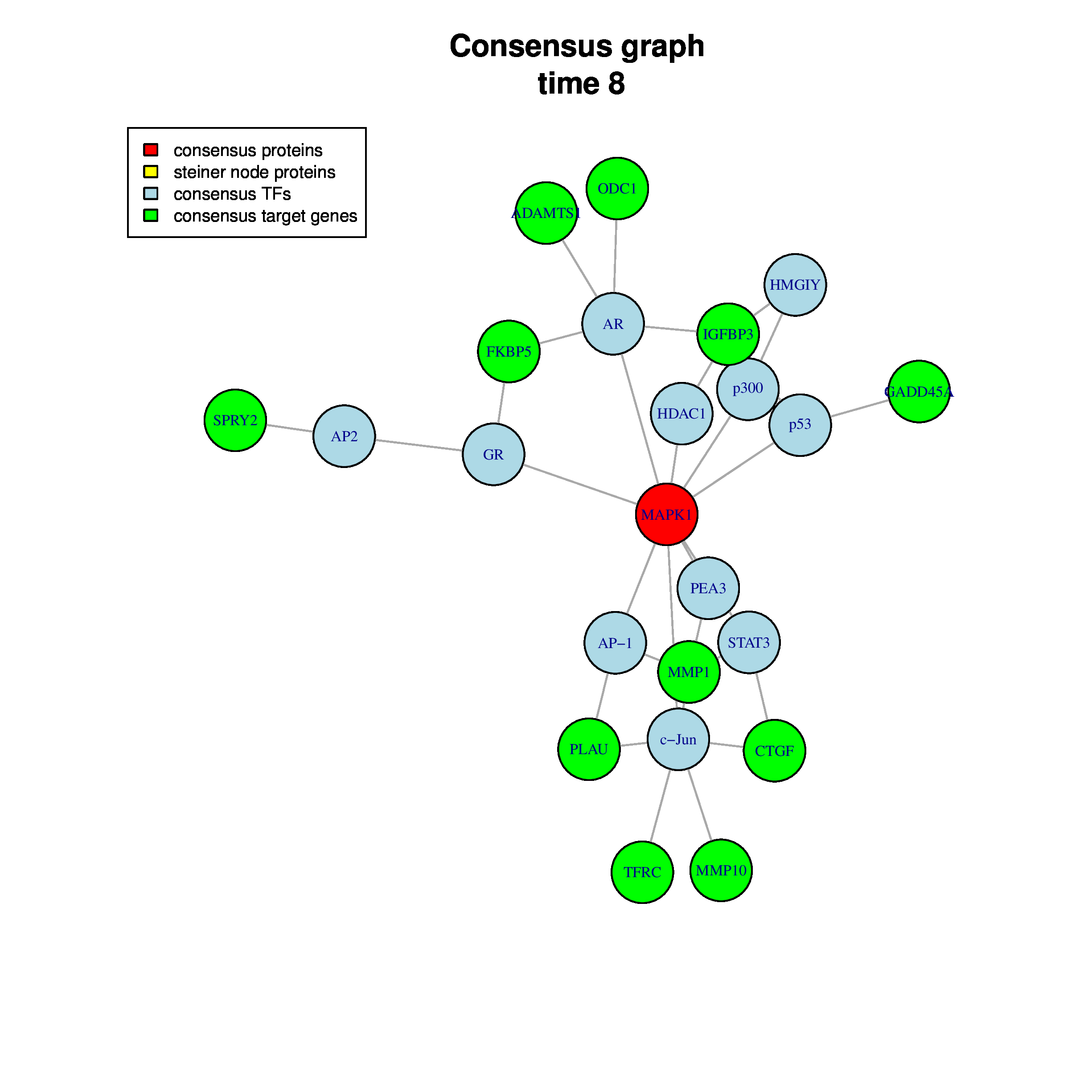

Supplement: Figure S4 — Static consensus graphs for time points 8 h after EGF stimulation. [file Image4.JPEG]

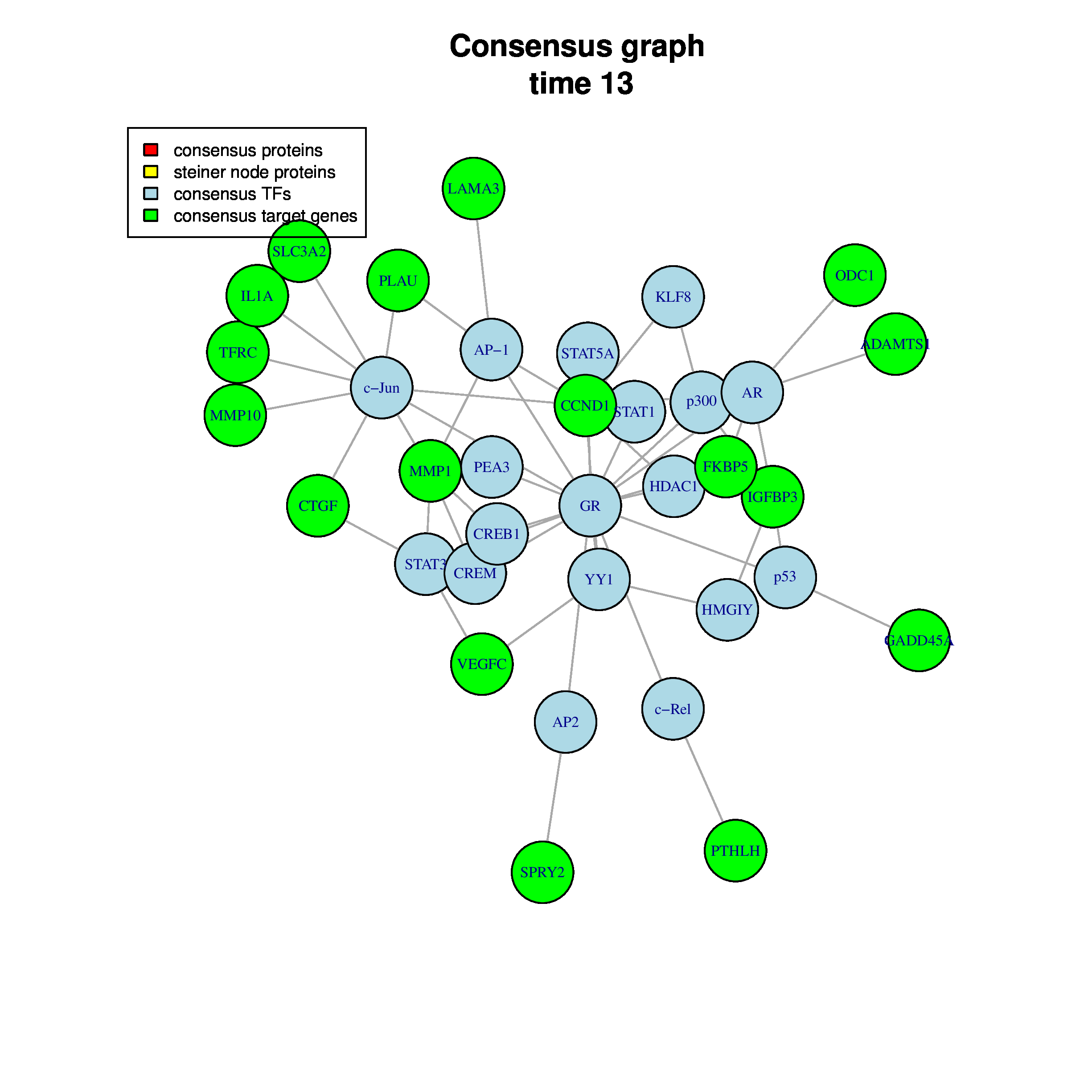

Supplement: Figure S5 — Static consensus graphs for time points 13 h after EGF stimulation. [file Image5.JPEG]

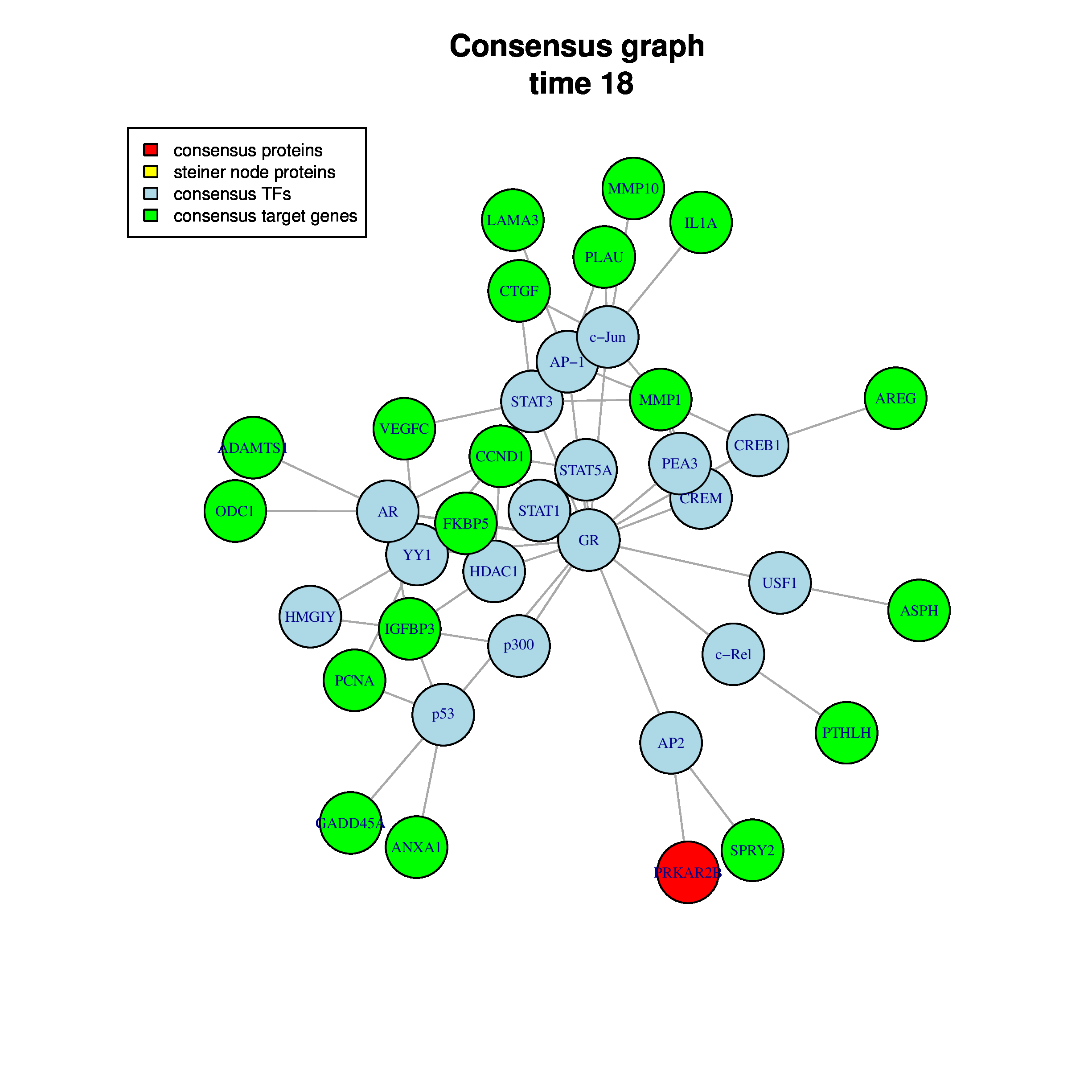

Supplement: Figure S6 — Static consensus graphs for time points 18 h after EGF stimulation. [file Image6.JPEG]

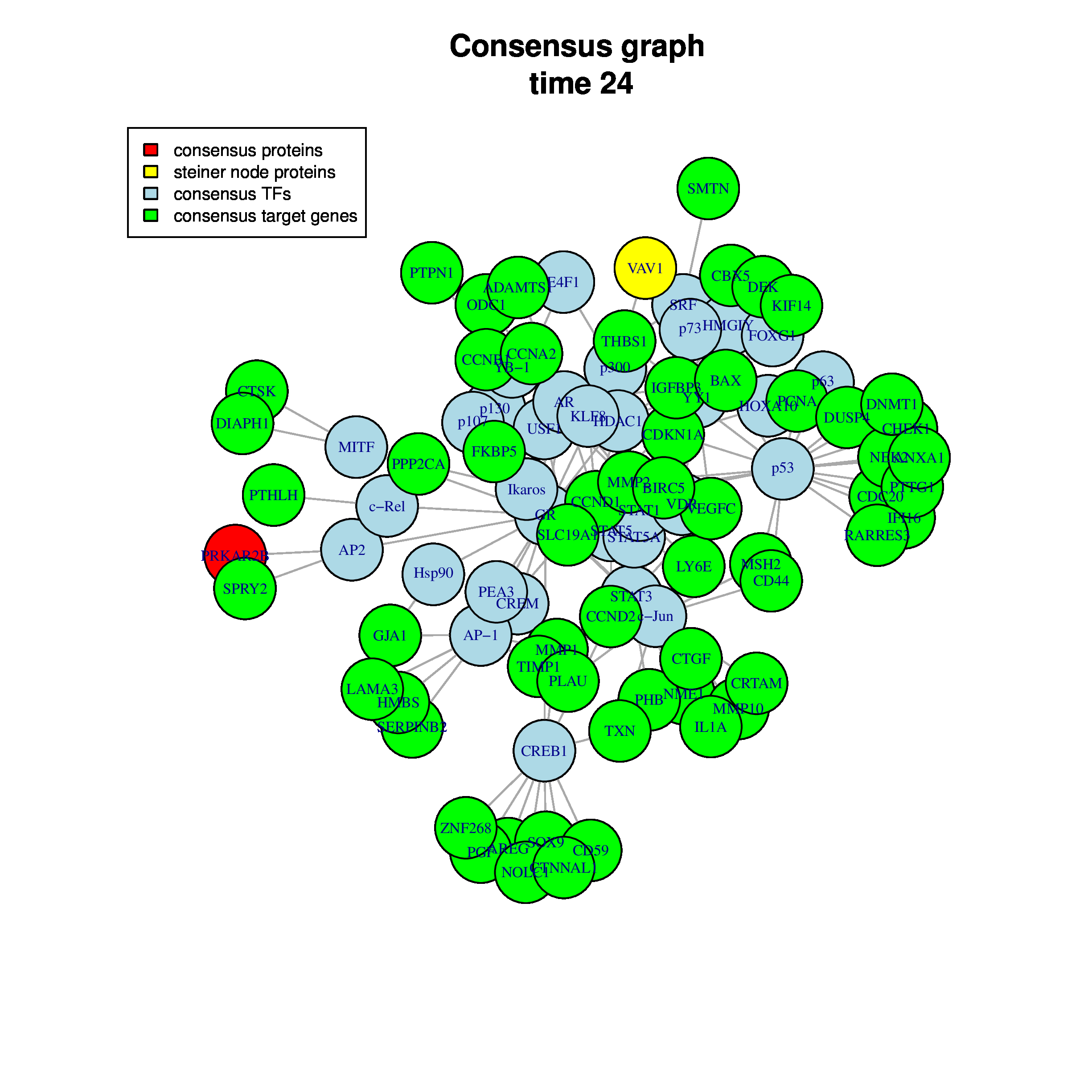

Supplement: Figure S7 — Static consensus graphs for time points 24 h after EGF stimulation. [file Image7.JPEG]

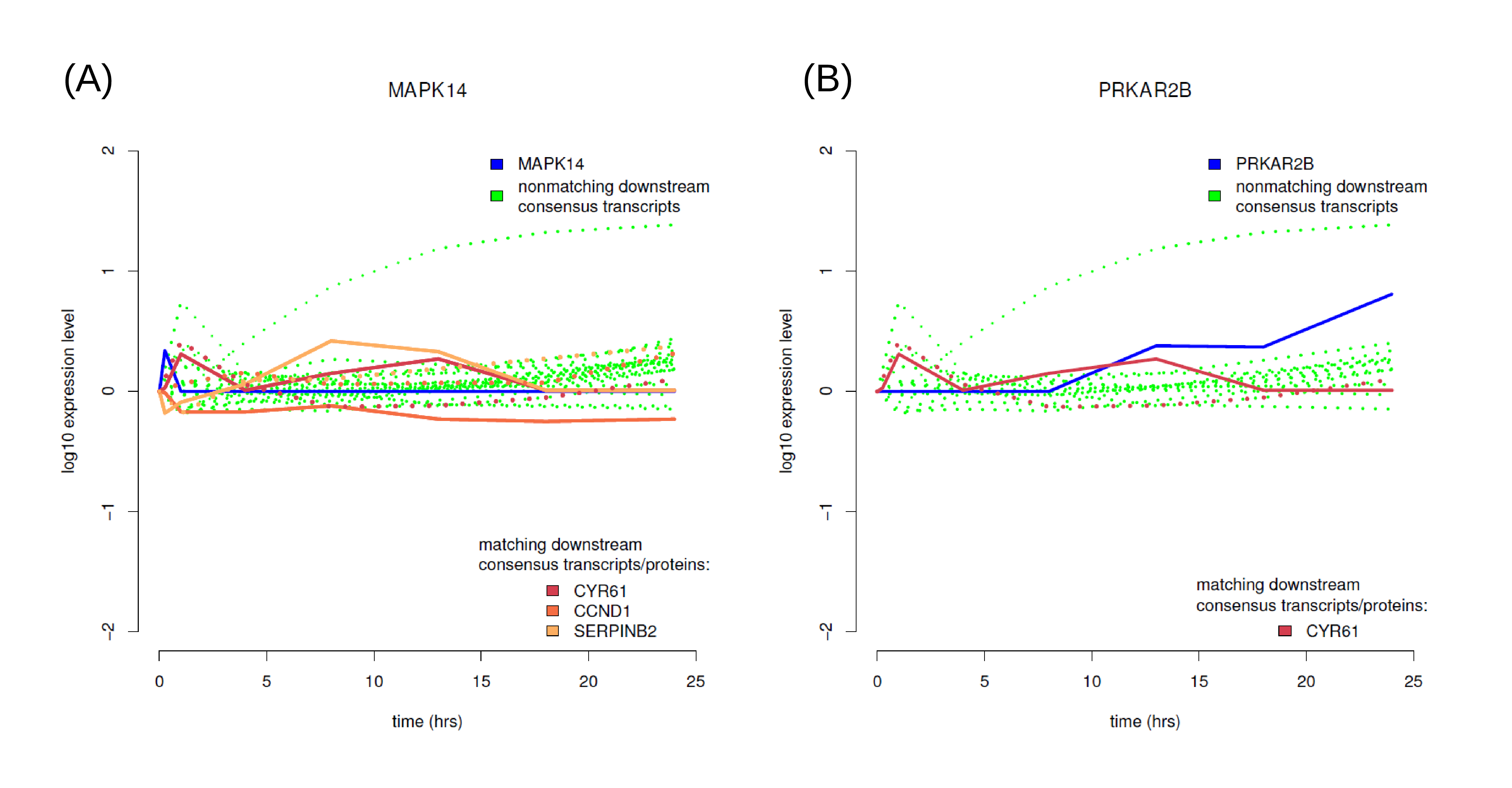

Supplement: Figure S8 — Time course integration for phosphoproteins MAPK14 and PRKAR2B. Downstream consensus transcripts identified for MAPK14 and PRKAR2B were mapped to differentially abundant proteins. Note that the measurement range of the expression profiles across platforms can vary. Phosphoprotein time course data is shown in solid, black lines, non-matching transcript data in solid, gray lines and matching transcript and proteome data in rainbow color palette with proteins depicted as solid lines and transcripts depicted as dotted lines. [file Image8.JPEG]
